# Crosstalk between SNF1 Pathway and the Peroxisome-Mediated Lipid Metabolism in *Magnaporthe oryzae*

**DOI:** 10.1371/journal.pone.0103124

**Published:** 2014-08-04

**Authors:** Xiao-Qing Zeng, Guo-Qing Chen, Xiao-Hong Liu, Bo Dong, Huan-Bin Shi, Jian-Ping Lu, Fucheng Lin

**Affiliations:** 1 State Key Laboratory for Rice Biology, Biotechnology Institute, Zhejiang University, Hangzhou, China; 2 State Key Laboratory of Rice Biology, China National Rice Research Institute, Hangzhou, China; 3 Institute of Virology and Biotechnology, Zhejiang Academy of Agricultural Science, Hangzhou, China; 4 College of Life Sciences, Zhejiang University, Hangzhou, China; 5 China Tobacco Gene Research Center, Zhengzhou Tobacco Institute of CNTC, Zhengzhou, China; University of Nebraska-Lincoln, United States of America

## Abstract

The SNF1/AMPK pathway has a central role in response to nutrient stress in yeast and mammals. Previous studies on SNF1 function in phytopathogenic fungi mostly focused on the catalytic subunit Snf1 and its contribution to the derepression of cell wall degrading enzymes (CWDEs). However, the MoSnf1 in *Magnaporthe oryzae* was reported not to be involved in CWDEs regulation. The mechanism how MoSnf1 functions as a virulence determinant remains unclear. In this report, we demonstrate that MoSnf1 retains the ability to respond to nutrient-free environment via its participation in peroxisomal maintenance and lipid metabolism. Observation of GFP-tagged peroxisomal targeting signal-1 (PTS1) revealed that the peroxisomes of *ΔMosnf1* were enlarged in mycelia and tended to be degraded before conidial germination, leading to the sharp decline of peroxisomal amount during appressorial development, which might impart the mutant great retard in lipid droplets mobilization and degradation. Consequently, *ΔMosnf1* exhibited inability to maintain normal appressorial cell wall porosity and turgor pressure, which are key players in epidermal infection process. Exogenous glucose could partially restore the appressorial function and virulence of *ΔMosnf1*. Toward a further understanding of SNF1 pathway, the β-subunit MoSip2, γ-subunit MoSnf4, and two putative Snf1-activating kinases, MoSak1 and MoTos3, were additionally identified and characterized. Here we show the null mutants *ΔMosip2* and *ΔMosnf4* performed multiple disorders as *ΔMosnf1* did, suggesting the complex integrity is essential for *M. oryzae* SNF1 kinase function. And the upstream kinases, MoSak1 and MoTos3, play unequal roles in SNF1 activation with a clear preference to MoSak1 over MoTos3. Meanwhile, the mutant lacking both of them exhibited a severe phenotype comparable to *ΔMosnf1*, uncovering a cooperative relationship between MoSak1 and MoTos3. Taken together, our data indicate that the SNF1 pathway is required for fungal development and facilitates pathogenicity by its contribution to peroxisomal maintenance and lipid metabolism in *M. oryzae*.

## Introduction

The conserved SNF1/AMP-activated protein kinase (AMPK) family is well known to serve as the cellular energy sensor and regulator of carbon metabolism in eukaryotes [1], [2], [3]. The yeast SNF1 kinase is a heterotrimer, composed of a catalytic α-subunit Snf1, a regulatory γ-subunit Snf4, and one of the three β-subunit isoforms, Sip1, Sip2, or Gal83, which tethers Snf1 and Snf4 together to form the functional kinase complex [4]. The best documented function of SNF1 kinase is to respond to glucose limitation and enable yeast cells to utilize non-preferred carbon sources when glucose is deprived [3], [5]. The kinase activity of SNF1 is activated by its upstream kinases, Sak1, Tos3, and Elm1 in yeast, which phosphorylate the activation-loop residue Thr210 of the Snf1/α subunit [6], [7]. Although Sak1 is the major kinase in this activation, only simultaneous absence of the three Snf1-activating kinases confers completely abolished growth on non-preferred carbon sources, indicating a partially redundant function among them [7], [8], [9]. One of the best-studied targets of yeast SNF1 is the transcriptional repressor Mig1, which represses the expression of pivotal enzymes involved in the utilization of alternative sugars [10]. Upon glucose depletion, SNF1 is activated by its upstream kinases and thereafter phosphorylates the repressor Mig1, resulting in the translocation of Mig1 from the nucleus to the cytoplasm and the relief of transcriptional repression imposed by Mig1 [3], [10]. Besides Mig1, SNF1 also regulates transcriptional activators, such as Cat8, Adr1, and Sip4, which activate the expression of many genes involved in peroxisome biosynthesis, gluconeogenesis, the glyoxylate cycle, as well as β-oxidation [11], [12], [13]. In addition to nutrient stress, the yeast SNF1 pathway also participates in environmental stress resistance, aging, invasive and pseudohyphal growth [3], [14], [15].

To date, most studies of Snf1 function in phytopathogenic fungi focused on its contribution to the derepression of cell wall degrading enzymes (CWDEs) [16], [17], [18], [19]. For plant pathogens, the major barrier of penetration to the host is plant cell wall. Many pathogenic fungi successfully overcome the obstacle by employing mechanical forces or enzymatic methods or a combination of both [19], [20]. Cell wall degrading enzymes (CWDEs), which can depolymerize the different constituents of plant cuticle, occupy an important position in pathogenesis [20]. Production of these enzymes is subject to carbon catabolite repression [21], and in some phytopathogenic fungi Snf1 is required for relieving such repression and upregulating CWDEs expression when invasion occurs [16], [17], [18], [19]. Removal of *SNF1* was reported to cause loss or significant reduction of pathogenicity in some plant pathogens, such as *Cochliobolus carbonum* [16], *Ustilago maydis* [19], and *Verticillium dahliae* [18]. The essential role of Snf1 imposed on virulence is partially attributed to its derepression of CWDEs, and another potential contributing factor is the Snf1-dependent utilization of alterative sugars, which can be acquired from the host to drive infection [16], [17], [18], [22]. However, the MoSnf1 in *Magnaporthe oryzae* is not involved in derepression of CWDEs or the metabolism of alterative sugars [23]. Therefore the key player employed by MoSnf1 in pathogenesis remains mysterious. Furthermore, except the catalytic subunit Snf1, the function of other components incorporated in the critical pathway has been rarely reported in filamentous fungi hitherto.

*M. oryzae*, a heterothallic ascomycete fungus, is the causal agent of the rice blast disease [24]. Upon recognition of environmental cues, the fungus differentiates a well-specialized cell structure, appressorium, from the end of a short germ tube after the contact and germination of a conidium on the host leaf [25]. During maturation, the appressorium becomes melanin-pigmented and accumulates substantial glycerol to generate hydrostatic turgor of up to 8 MPa through vacuolar degradation of lipid reserves [26]. Relying on the turgor pressure, the fungus elaborates a penetration peg to mechanically punch the plant cuticle, and subsequently colonizes host tissues [27]. Since the differentiation of infection structures is in an environment without exogenous nutrients, it underlines the fact that the early stages of plant infection are fuelled by compounds reserved in the conidium. At the onset of appressorial development, translocation of mass lipid bodies from conidium to appressorium occurs, accompanied by rapid lipolysis in appressorial vacuole. Triglycerides, the most abundant form of lipids, are degraded to fatty acids and glycerol under the catalysis of triacylglycerol lipases [26], [28]. Consequently, a requirement for fatty acid β-oxidation and subsequent activation of the glyoxylate cycle and gluconeogenesis has been proposed [26], [28], [29]. The fatty acid β-oxidation, occurred predominantly in peroxisome, leads to the generation of acetyl-CoA pool, which is available for melanin biosynthesis pathway and also fuels fungal cell wall biosynthesis via the glyoxylate bypass and gluconeogenesis during plant infection [30], [31], [32]. The importance of such process is highlighted by the fact that mutants impaired in peroxisome biosynthesis [30], [33], [34], β-oxidation [35], carnitine acetyl transfer system [31], [36], or glyoxylate cycle [29] are non-pathogenic. Meanwhile, recent transcriptomic analysis independently confirms the pivotal role of peroxisomal acetyl-CoA production during appressorial development [37]. In yeast, both lipid metabolism and peroxisomal proliferation are under the regulation of SNF1 pathway [5], [38]. As opposed to the wealthy information in yeast, the function of SNF1 in these critical processes has not yet been studied in filamentous fungi.

In this study, the *M. oryzae* SNF1 pathway was systematically characterized by targeted deletions of the three SNF1 complex subunits and two putative upstream Snf1-activating kinases. Through investigation of GFP-PTS1 signals, we found SNF1 pathway is indispensable for peroxisomal maintenance, which might account for its essential role in lipid metabolism. Furthermore, the interruption of SNF1 pathway resulted in enlarged size of appressorial wall pore and decreased turgor pressure, ultimately the loss of pathogenicity. Our results highlight the importance of SNF1 complex integrity, the upstream kinases, and their contributions to energy homeostasis.

## Results

### Identification of the SNF1 complex components and two putative Snf1-activating kinases in *M. oryzae*

**Figure 1:**
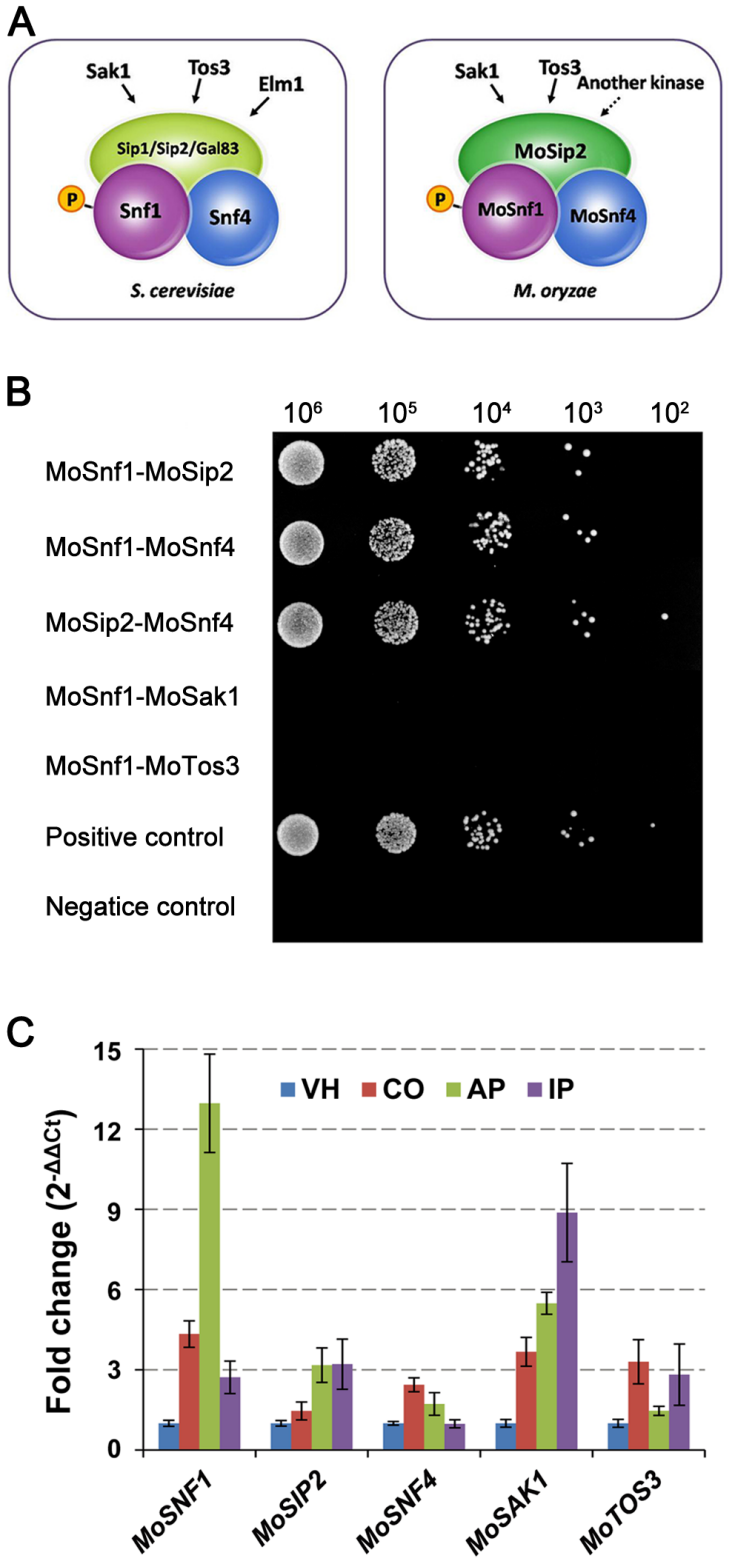
Protein interaction and gene expression analyses of SNF1 kinase complex components and its activating kinases in *M. oryzae*. (**A**) Different composition of the heterotrimeric SNF1 kinase complex and upstream kinases between *S. cerevisiae* and *M. oryzae*. (**B**) MoSnf1, MoSip2, and MoSnf4 interacted with each other, while no interaction was observed between MoSnf1 and its activating kinases in yeast two-hybrid assay. Yeast transformants expressing MoSnf1 plus MoSip2, MoSnf1 plus MoSnf4, MoSip2 plus MoSnf4, MoSnf1 plus MoSak1, or MoSnf1 plus MoTos3 were 10-fold serially diluted with a starter culture of 10^6^ cells/ml and then spotted (5 µl) onto SD-Trp-Leu-His-Ade medium. (**C**) Gene expression profiles of *MoSNF1*, *MoSIP2*, *MoSNF4*, *MoSAK1*, and *MoTOS3* among different developmental stages. Tested fungal tissues included vegetative hyphae (VH), conidia (CO), appressoria 8 hpi (AP), and invasive hyphae (72 hpi), which were within infected plant leaves (IP). Gene expression data, obtained from quantitative RT-PCR analysis, were normalized by using β-tubulin as an internal control and calibrated against the transcript abundances of VH stage.

In *Saccharomyces cerevisiae*, the SNF1 kinase complex consists of three subunits, the catalytic subunit Snf1, the γ-subunit Snf4, and one of the three β-subunit isoforms, Gal83, Sip1, or Sip2 [4]. There are three upstream Snf1-activating kinases (Sak1, Elm1, and Tos3), each of which is sufficient to activate the SNF1 complex. MoSnf1 (MGG_00803), the catalytic subunit of SNF1 complex in *M. oryzae*, had been identified and proved functionally homologous to *S. cerevisiae* Snf1 [23]. In this study, we additionally sought for other *M. oryzae* orthologs involved in SNF1 pathway to obtain a further understanding of its function. Using protein sequences of *S. cerevisiae* counterparts for BLASTP searches, we identified only one β subunit MoSip2 (MGG_06930), the γ subunit MoSnf4 (MGG_04005), and two upstream kinases, MoSak1 (MGG_07003) and MoTos3 (MGG_06421) in the *M. oryzae* genome (http://www.broadinstitute.org/annotation/genome/magnaporthe_comparative/MultiHome.html), named after the best match (Figure 1A and Table S1). Domains identified by the InterPro database (http://www.ebi.ac.uk/interpro/) of these *M. oryzae* proteins exhibited high conservation (Table S1), including GBD (glycogen-binding domain) and ID (kinase interaction domain) in the C-terminal region of MoSip2, two pairs of cystathionine-beta-synthase (CBS) repeats integrated in MoSnf4, and kinase domains in MoSak1 and MoTos3.

Yeast two hybrid (Y2H) assays were carried out to clarify the interacting network of SNF1 pathway in *M. oryzae*. The results provided evidence that MoSnf1, MoSip2, and MoSnf4 physically interacted with each other, hinting the importance of the complex integrity (Figure 1B). Whereas no interaction was observed between MoSnf1 and MoSak1 or MoTos3 in Y2H (Figure 1B), unlike the stable association between Snf1 and Sak1 in yeast [39]. The result may be caused by the reason that the interaction between MoSnf1 and its upstream kinases was too transient to detect or only occurred under certain conditions.

### The expression profiles of SNF1 pathway components in *M*. *oryzae*

To obtain some insights into the potential function of SNF1 pathway components, gene expression patterns of the upstream Snf1-activating kinases (*MoSAK1* and *MoTOS3*) and SNF1 complex subunits (*MoSNF1*(α), *MoSIP2*(β), and *MoSNF4*(γ)), were examined by quantitative real-time RT-PCR (qRT-PCR) in vegetative hyphae, conidia, appressoria (8 hpi), and infected barley leaves (72 hpi). When normalized by the vegetative growth stage, the transcription of all the tested genes was up-regulated, although with varied up-regulation folds (Figure 1C). In comparison with the moderate increases of the two regulatory subunits, *MoSIP2* and *MoSNF4*, the expression levels of *MoSNF1* were greatly induced in conidia (4.34-fold) and appressoria (12.97-fold), suggesting a key role of MoSnf1 in the kinase complex. While during invasive growth, the transcription levels of *MoSNF1* and *MoSIP2* were elevated similarly, which was consistent with the observation that both were essential for pathogenicity (see below). Except similar transcript abundance in conidia, *MoSAK1* had much higher expression level increase than *MoTOS3* in appressoria and infected barley leaves, indicating the predominant MoSnf1-regulating position of MoSak1 over MoTos3. The SNF1 pathway up-regulation profile was indicative of its wide influence upon pathogenesis-related processes.

### The critical role of SNF1 complex integrity and its upstream kinases in conidiogenesis, aerial hyphae development, appressorial formation and morphology of *M. oryzae*

To further study the biological function of SNF1 pathway in *M. oryzae*, five null mutant strains specific to *MoSNF1*, *MoSIP2*, *MoSNF4*, *MoSAK1*, and *MoTOS3* were generated and verified by Southern blot analysis (Figure S1). Double deletion mutant *ΔMosak1ΔMotos3* was also constructed to determine whether the two upstream kinases have functional overlap. Finally, we acquired at least two independently targeted gene deletions for each mutant (*ΔMosnf1*, *ΔMosip2*, *ΔMosnf4*, *ΔMosak1*, *ΔMotos3*, and *ΔMosak1ΔMotos3*), and selected one strain for detailed phenotypic analysis.

**Figure 2:**
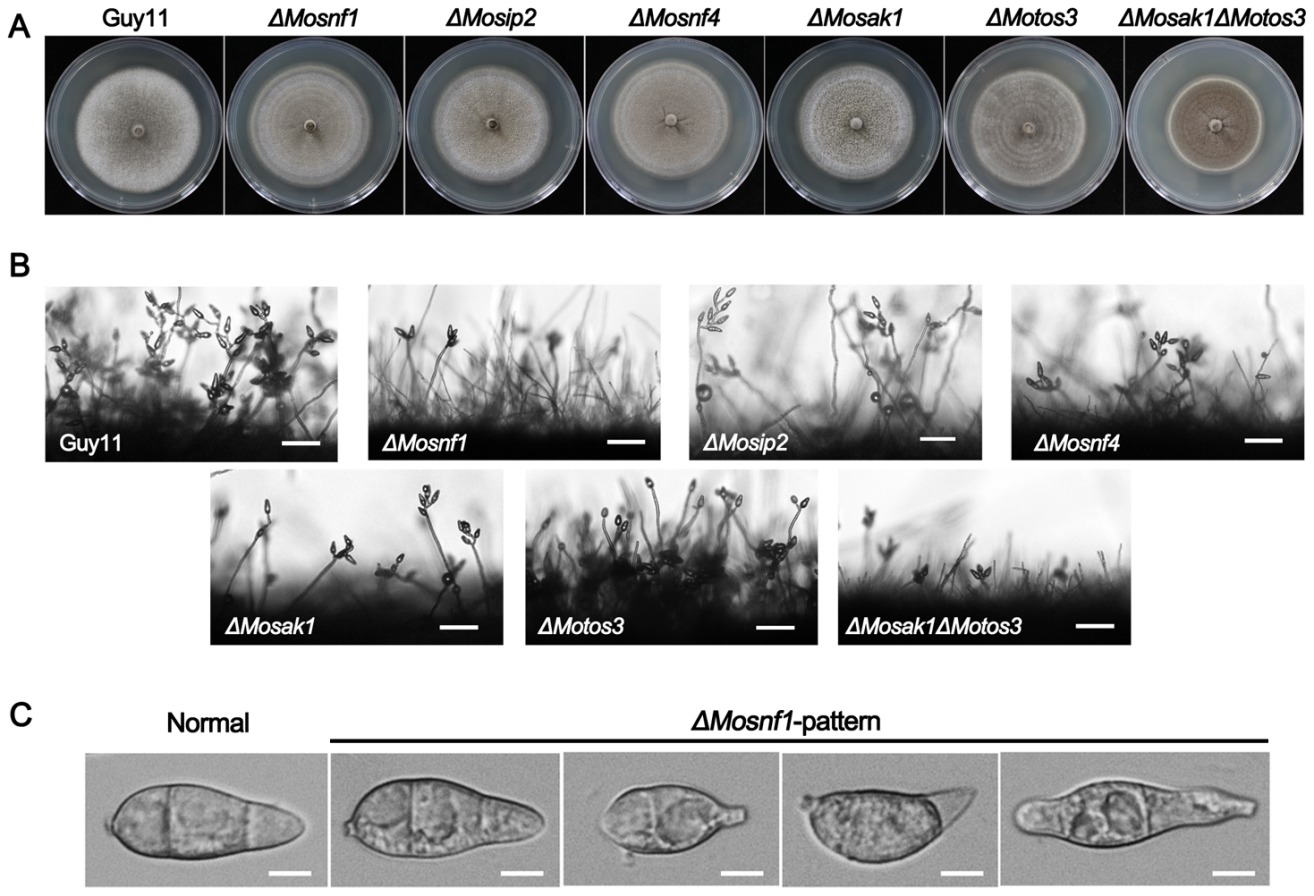
Comparison of the SNF1 pathway mutants with regard to colony morphology and conidial development. (**A**) Strains were cultured on CM plates at 25°C for 10 days. *ΔMosak1ΔMotos3* exhibited a decreased mycelial growth rate, while no significant difference in the colony size was observed between other mutants and Guy11. (**B**) Microscopic observation of conidial development. Significant reduction in conidial production was observed in *ΔMosnf1*, *ΔMosip2*, *ΔMosnf4*, *ΔMosak1*, and *ΔMosak1ΔMoto3* at 24 hpi. However, *ΔMotos3* developed short, yet dense conidiophores with plenty of spores arrayed thereon. Bars  = 50 µm. (**C**) Conidia of WT and the mutants were harvested and observed under the light microscope. Conidial shape of *ΔMosip2*, *ΔMosnf4*, *ΔMosak1*, and *ΔMosak1ΔMoto3* was identical to that of *ΔMosnf1* (*ΔMosnf1*-pattern), whereas there was no measurable difference between *ΔMotos3* and Guy11 (Normal). Bars  = 5 µm.

**Figure 3:**
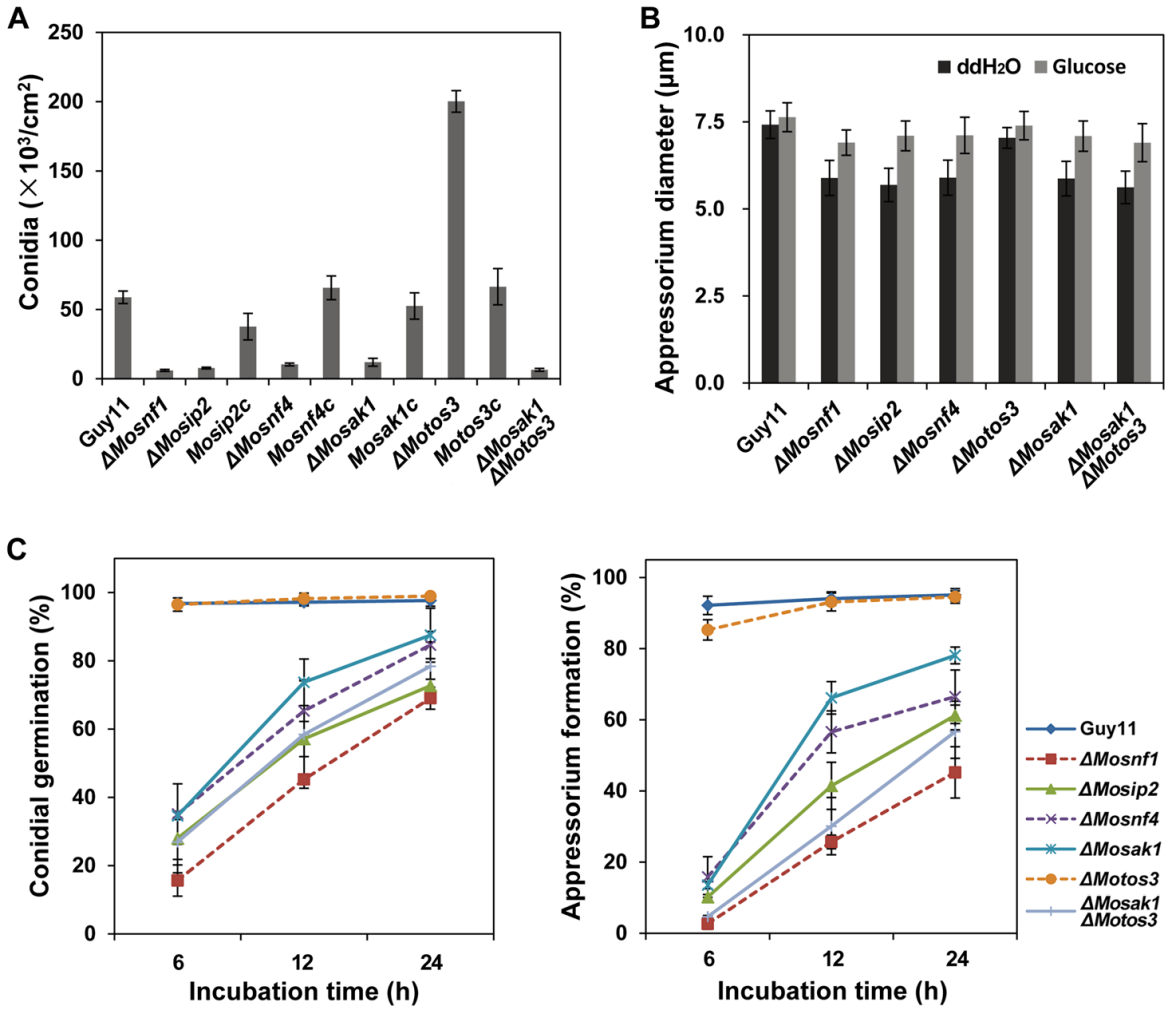
SNF1 pathway mutants exhibited dramatic defects in sporulation, appressorial size, conidial germination, and appressorium formation. (**A**) Conidial production of *ΔMosnf1*, *ΔMosip2*, *ΔMosnf4*, *ΔMosak1*, and *ΔMosak1ΔMoto3* was severely impaired, while *ΔMotos3* showed an elevated conidiation in comparison to Guy11. The complemented strains restored the sporulation defects of the corresponding mutants. Conidia were collected and counted from 10-day-old cultures grown on CM plates. The means and standard deviations of three independent experiments are presented as columns with error bars. (**B**) Appressorial diameters were measured and statistically analyzed after 48 h incubation on the artificial surfaces. Addition of 2.5% glucose to conidial suspensions partially restored the mutant defect in appressorial size. (**C**) Disruption of SNF1 pathway genes impaired conidial germination and appressorium formation in *M. oryzae*. Conidial suspensions harvested from 8-day-old CM cultures were incubated on hydrophobic surfaces and observed under a light microscope at indicated time points.

In brief, the morphological phenotypes of SNF1 complex mutants (including *ΔMosnf1*, *ΔMosip2*, and *ΔMosnf4*), were similar to each other, including abnormal spore appearance (Figure 2C), poor sporulation (Figure 2B and Figure 3A), reduced appressorial size (Figure 3B), shortened aerial hyphae (Figure 2A and 2B), but normal growth rate (Figure 2A) in complete media as compared to WT (wild-type) and complemented strains. Furthermore, all the three mutants exhibited delayed conidial germination and appressorial formation, the rates of which were significantly lower than WT strain even the incubation time was prolonged to 24 h (Figure 3C). Nevertheless, the defects in *ΔMosip2* and *ΔMosnf4* were more alleviated than that in *ΔMosnf1*.

The two upstream kinase mutants, *ΔMosak1* and *ΔMotos3*, exhibited different phenotypes during fungal development. *ΔMosak1* possessed sparse aerial hyphae with extremely poor conidiation, which was decreased by 5-fold compared to WT and the complemented strain (Figure 2B and Figure 3A). Besides, the process of spore germination and appressorial formation was greatly affected (Figure 3C). In contrast, the other upstream kinase mutant, *ΔMotos3*, formed much denser aerial hyphae with short branch, producing large scales of normal spores as many as 3-fold of WT (Figure 2 and Figure 3A). Re-introduction of *MoTOS3* into the mutant recovered these phenotypic changes. Whereas other phenotypes of *ΔMotos3* were indistinguishable from those of the wild-type strain (Figure 3B and 3C). In *ΔMosak1ΔMotos3* double mutant, the colony had an almost flat appearance, due to the attenuated aerial hyphae, and a slower growth rate (Figure 2A and 2B). The conidiogenesis and appressorial formation defects of the double mutant were more severe than *ΔMosak1* and comparable to the null SNF1 complex mutants (Figure 3), indicative of a collaborative relationship between MoSak1 and MoTos3 on the regulation of the SNF1 complex.

These results suggest that the SNF1 complex integrity is essential for aerial hyphae development, conidiogenesis, appressorial formation and morphology in *M. oryzae*. Meanwhile, the two upstream Snf1-activating kinases, MoSak1 and MoTos3, play distinct roles during above processes.

### Disruption of SNF1 pathway suppressed the mycelial growth on media with non-fermentable carbon sources

In yeast, *Δsnf1* is unable to survive on media with non-fermentable carbon as sole carbon source [5]. To determine whether *M. oryzae* SNF1 pathway contributes to non-fermentable carbon metabolism, mycelial agar plugs of the SNF1 pathway mutants were incubated on minimal media supplemented with various non-fermentable carbons as sole carbon source.

**Figure 4:**
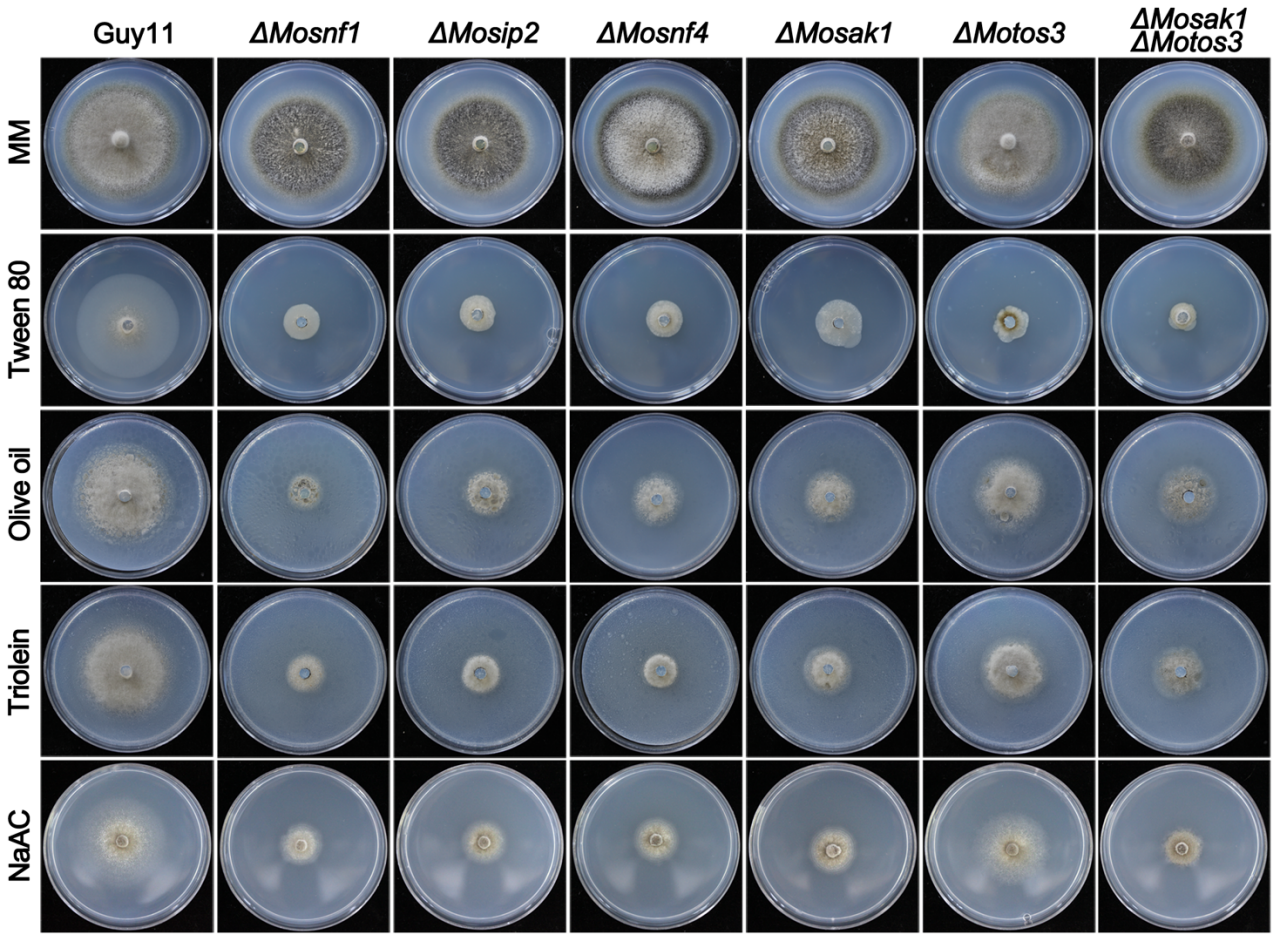
Mutations in SNF1 pathway affected the utilization of non-fermentable carbons. Strains were cultured on MM plates supplemented with 1% Glucose, 1% Tween 80, 1% Olive oil, 1% Triolein, or 50 mM Sodium acetate as sole carbon source for 10 d at 25°C.

Growth tests revealed that all the tested mutants, except *ΔMotos3*, exhibited severe defects in utilization of acetate, Tween 80 (the principal component is oleate), triolein (one of the typical triglycerides), and olive oil (long chain fatty acids) (Figure 4 and Table 1). The growth rate of *ΔMotos3* was slightly affected except on Tween 80-contained medium (Figure 4 and Table 1). Among other mutants, the deficiency degree varied upon carbon type. Overall, the defects of *ΔMosip2* and *ΔMosnf4* were slightly relieved as compared to *ΔMosnf1* (Figure 4 and Table 1). The mycelial growth of *ΔMosak1ΔMotos3* was comparable to *ΔMosak1* except on Tween 80 medium, with 88% and 75% growth reduction, respectively (Figure 4 and Table 1). These data indicate that the SNF1 pathway is of great importance to the efficient utilization of non-fermentable carbon sources in *M. oryzae.*

**Table 1:** Growth rate of the SNF1 pathway mutants on non-fermentable carbon media.

Strain	Olive oil	Tween-80	Triolein	NaAC
Guy11	76.5±2.4^a^	70.0±2.2^a^	78.4±0.7^a^	69.5±3.1^a^
*ΔMosnf1*	21.5±3.1^d^	18.2±0.6^c^	23.3±0.6^e^	24.6±2.1^d^
*ΔMosip2*	31.3±0.6^c^	17.2±0.6^cd^	25.4±1.1^de^	27.7±2.2^cd^
*ΔMosnf4*	34.0±2.3^c^	18.9±1.3^c^	29.8±6.2^cd^	26.9±1.2^cd^
*ΔMosak1*	34.7±3.3^c^	25.3±1.6^b^	30.6±0.6^cd^	31.0±0.8^c^
*ΔMotos3*	53.5±1.8^b^	13.8±3.4^de^	50.8±3.1^b^	56.3±4.7^b^
*ΔMosak1ΔMotos3*	35.6±2.4^c^	11.9±4.0^e^	32.2±2.4^c^	26.5±0.8^cd^

Vegetative growth rate (%)  =  (the diameter of strains on MM with various carbon sources/the diameter of cultures on regular MM plates) ×100; colony diameters on regular MM plates are set as 100% control. Data were collected from 10 days postincubation and presented as means±SD from three independent experiments. Duncan's multiple range tests were used to determine significance at the 0.05 level of probability. The same letters in a column mean no significant difference.

### The maintenance of peroxisomes is dependent on the SNF1 pathway

The single-membrane organelle peroxisome is well known for its function in fatty acid β-oxidation, the glyoxylate cycle, and removal of reactive oxygen species (ROS) [40]. In *M. oryzae*, mutants with abnormal peroxisomes such as *ΔMopex6* [30], *ΔMopex5* [34], *ΔMopex7* [33], and *ΔMopex19* [41] showed inability to metabolize fatty acids and NaAC. In addition, yeast *Δsnf1* and *Δsnf4* were reported to be devoid of peroxisomal structures [38]. Thus, we carried out to assess the association between SNF1 pathway and peroxisomal biogenesis in *M. oryzae*.

**Figure 5:**
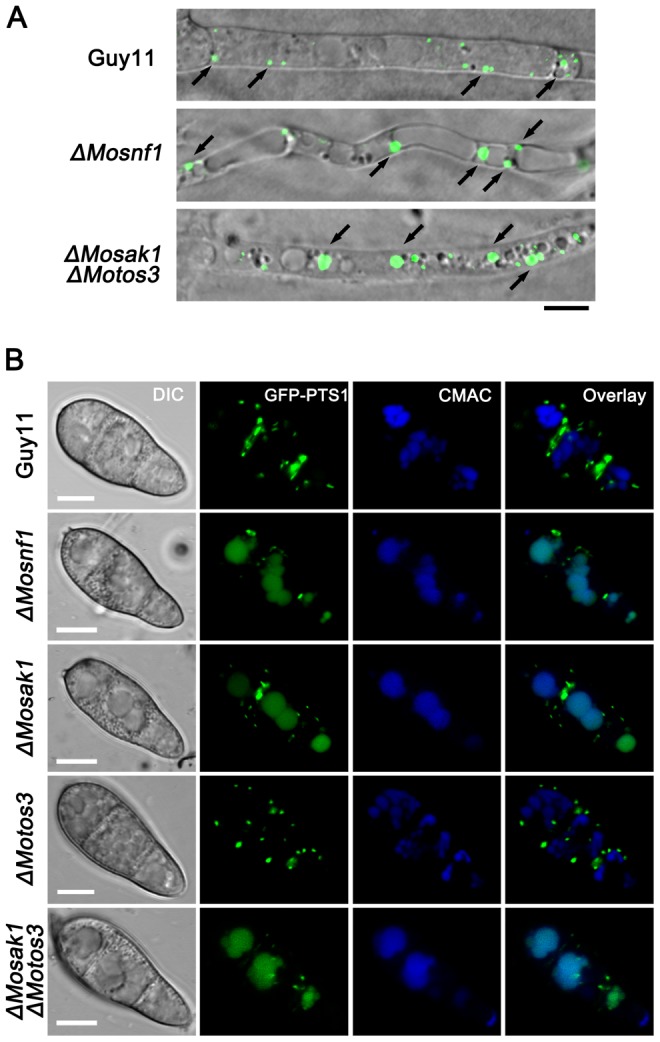
Effects of SNF1 pathway mutations on GFP-PTS1 distribution. (**A**) Confocal microscopic observation of mutant strains expressing GFP-PTS1. Images shown were representative of the majority of vegetative hyphae. Enlarged peroxisomes were more frequently observed in *ΔMosnf1* and *ΔMosak1ΔMotos3* than WT. Arrows point to peroxisomes. Bar  = 5 µm. (**B**) Colocalization of GFP-PST1-positive peroxisomes and CMAC-stained vacuoles. The amount of cytoplasmic peroxisomes was decreased dramatically in the conidia of *ΔMosnf1*, *ΔMosak1*, and *ΔMosak1ΔMotos3*, while in WT and *ΔMotos3*, numerous peroxisomal puncta were observed with the absence of vacuolar GFP fluorescence. The localization patterns of GFP-PTS1 in *ΔMosip2* and *ΔMosnf4* conidia were indistinguishable from that in *ΔMosnf1* conidia. Bars  = 5 µm.

Peroxisomal matrix proteins usually include specific motifs known as peroxisomal targeting signals (PTSs), which could be recognized by the import machinery and targeted to peroxisome. PTS1 is a conserved tripeptide sequence (S/A/C) (H/R/K) (I/L/M) at the C terminus of most known peroxisomal matrix proteins [34], [40]. In this study, PTS1 (SKL) signal was employed to visualize peroxisome by introducing GFP-PTS1 vector (kindly provided by Dr. Jiaoyu Wang) into *ΔMosnf1*, *ΔMosip2*, *ΔMosnf4*, *ΔMosak1*, *ΔMotos3*, *ΔMosak1ΔMotos3*, and the wild type, respectively. Subcellular localization of GFP-PTS1 was then investigated in the transformed strains. During vegetative growth phase, both Guy11 and the SNF1 pathway mutants performed punctate GFP fluorescence, indicative of peroxisomal structures, however, the puncta size seemed to be different among them. In *ΔMosnf1*, *ΔMosip2*, *ΔMosnf4*, *ΔMosak1*, and *ΔMosak1ΔMotos3* background, enlarged peroxisomes were more frequently observed than in WT and *ΔMotos3*, suggesting some aberrant changes therein (Figure 5A). Likewise, numerous GFP-PTS1 labeled peroxisomes were observed as punctate spots in the conidia of WT and *ΔMotos3* (Figure 5B), and began to enter the vacuolar lumens during appressorial differentiation (Figure S2); in the meantime, their incipient appressoria were peroxisome-rich (Figure S2). While in other mutants, GFP-PTS1 had already been mis-localized to the CMAC-stained vacuoles even before conidial germination, leaving sharp decline of fluorescent spots in the conidial cytoplasm (77.6±5.1%, 64.3±4.6%, 62.2±5.7%, 35.5±5.3%, 76.9±6.6% spores with fluorescent vacuoles in *ΔMosnf1*, *ΔMosip2*, *ΔMosnf4*, *ΔMosak1*, and *ΔMosak1ΔMotos3*, respectively, vs. 1.6±0.7%, 1.4±0.6% in WT and *ΔMotos3*, respectively) (Figure 5B). Further investigation of the GFP-PTS1 sequential localization revealed that the fluorescent signals were almost invisible in *ΔMosnf1* appressoria but arrested in conidial spherical vacuoles (Figure S2).

These findings indicate that the *M. oryzae* SNF1 pathway plays an important role in peroxisomal dynamics during fungal development.

### Lipid mobilization was dramatically retarded in *ΔMosnf1*, *ΔMosip2*, *ΔMosnf4*, *ΔMosak1*, and *ΔMosak1ΔMotos3*

During appressorial development, lipid reserves in conidia are rapidly transferred to appressoria where they are degraded and supplied as the source of glycerol and other intermediates, a process demanding peroxisomal function [28], [35]. To determine whether the impairment of peroxisomal maintenance affected the lipid droplets mobilization in the SNF1 pathway mutants, lipid bodies were monitored during appressorial morphogenesis via Nile red staining.

**Figure 6:**
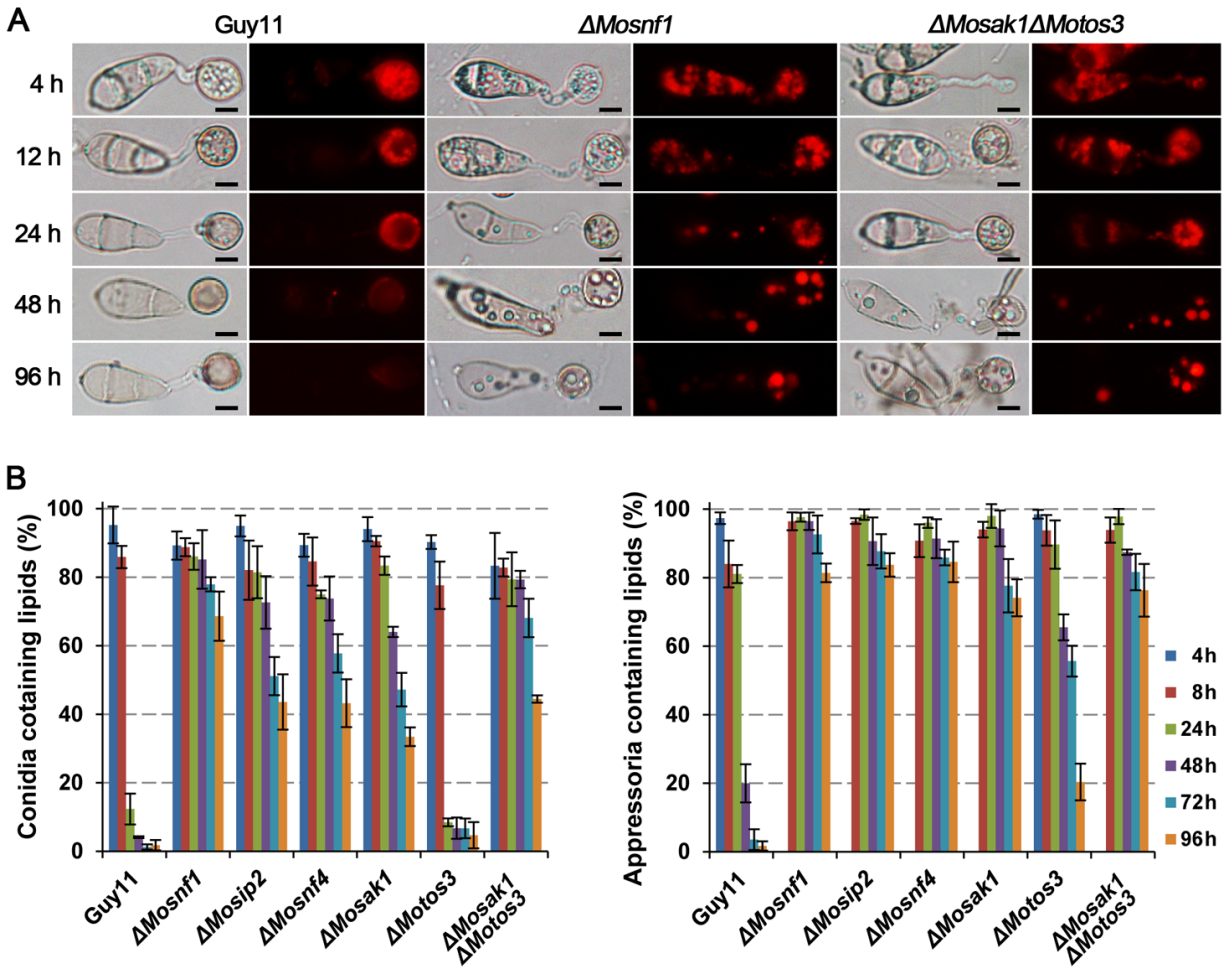
Intracellular mobilization of lipid droplets in WT and SNF1 pathway mutants during appressorium morphogenesis. Conidial suspensions were incubated on the surfaces of hydrophobic films and stained with Nile red to observe the status of lipid droplets movement and distribution at the indicated time points under epifluorescence microscope. (**A**) *ΔMosnf1* and *ΔMosak1ΔMotos3* showed significant delays in lipid mobilization and degradation with the presence of Nile red-stained lipid bodies even at 96 hpi, while fluorescent signals were almost invisible in WT at 48 hpi. Bars  = 5 µm. (**B**) Percentages of conidia (left) or appressoria (right) that contained lipid droplets. Varied degrees of defect in lipid mobilization were observed among the mutants.

It was observed that lipid droplets had been entirely translocated from conidia to appressoria within 24 h and the majority was degraded at 48 hpi in Guy11 (Figure 6). In *ΔMotos3*, no distinguishable difference was detected in the transfer efficiency of lipid bodies as compared to WT, but the degradation rate was slightly decreased, with about 20% appressoria stained by Nile red compared to only 1.67% in Guy11 at 96 hpi (Figure 6B). On the contrary, the mobilization of lipid reserves was significantly retarded in *ΔMosnf1*, *ΔMosip2*, *ΔMosnf4*, *ΔMosak1*, and *ΔMosak1ΔMotos3*, ranging from 64.0% to 85.2% conidia still containing large lipid deposits after 48 h of incubation (Figure 6A, 6B left). The degradation rates were also severely influenced, with large merged lipid droplets still observed in more than 74% appressoria of the mutants at 96 hpi (Figure 6A, 6B right). We therefore conclude that the SNF1 pathway is indispensible for lipid droplets translocation and degradation.

### SNF1 pathway is responsible for turgor genesis and normal porosity of the appressorial wall

**Figure 7:**
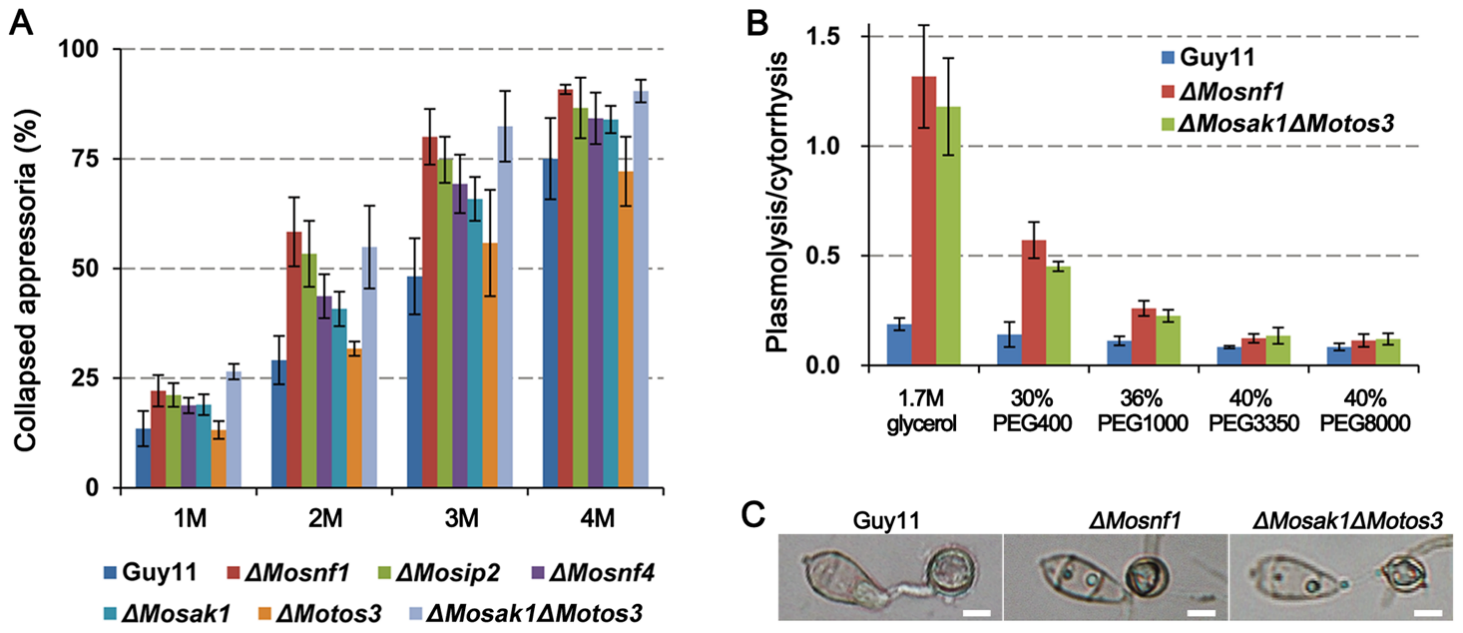
Indirect assessment of turgor pressure and appressorial porosity. (**A**) To measure appressorial turgor pressure, incipient cytorrhysis assay was performed on induced appressoria at 48 hpi with glycerol solutions of varying concentrations (1–4 M). (**B**) Plasmolysis/cytorrhysis assay with osmotic solutions of different average molecular weights. The solutions were adjusted to the denoted concentrations to exert 4 MPa osmotic pressure on appressoria at 48 hpi. (**C**) Plasmolyzed appressoria at 48 hpi were photographed after soaked in 1.7 M glycerol solution for 10 min. Bars  = 5 µm.

Appressorium-mediated penetration in *M. oryzae* is a turgor-driven process required substantial glycerol accumulation. The predominant cellular source of glycerol is from lipid droplets degradation during appressorium maturation [31], [42]. Since SNF1 pathway participates in the degradation of lipid bodies, cytorrhysis assay [43] was performed to test whether the SNF1 pathway mutants had the ability to generate turgor pressure comparable to WT. The appressorial collapse rates of Guy11 and *ΔMotos3* were similar under a serial concentrations of glycerol (from 1 M to 4 M) (Figure 7A), while the appressoria of the other mutants were much more fragile and vulnerable to collapse, with the severity from high to low as follows: *ΔMosnf1*/*ΔMosak1ΔMotos3*, *ΔMosip2*, *ΔMosnf4*, and *ΔMosak1* (Figure 7A). So the impairment in SNF1 function dose affect the accumulation of appressorial turgor pressure.

When dipped in glycerol solution, plasmolysis was frequently observed in the appressoria of the mutants. Thus we speculated the SNF1 pathway might also play a key role in appressorial cell wall integrity. To explore the possibility, cytorrhysis/plasmolysis test was carried out by applying solutions of polyethylene glycols (PEGs) with different molecular weights but the same osmotic pressure (4 MPa) to appressoria at 48 hpi. Through calculating the ratio of plasmolysis to cytorrhysis, we found for the wild type the proportion of appressoria showing cytorrhysis was dominant over all the tested PEG types (Figure 7B). However, in *ΔMosnf1* and *ΔMosak1ΔMotos3*, the ratio of plasmolysis/cytorrhysis declined along with the diameter of external solute species increased, and was eventually similar to that of WT in PEG3350 or larger (Figure 7B). Furthermore, the plasmolysis was more severe in mutants than WT as shown in Figure 7C. These data suggest the SNF1 function is essential for maintaining proper porosity of the appressorial wall. Transmission electron microscopy (TEM) was also performed to observe the appressorial structures in detail. As a result, electron-dense melanin layer, with identical thickness, was distinctly observed in both wild type and the mutants (Figure S3), suggesting the appressorial wall defects of the SNF1 pathway mutants are not associated with melanin layer biosynthesis.

Lipid degradation not only liberates glycerol but also feeds the acetyl-CoA pool produced by beta-oxidation of fatty acids. The end product acetyl-CoA can be shuttled to the glyoxylate cycle and gluconeogenesis which enable it to synthesize components of cell wall such as glucans and chitin [31], [32]. Hence, we inferred why the SNF1 pathway mutants failed to maintain sufficient turgor pressure was caused by the reduced accumulation of intracellular osmolites and increased appressorial cell wall porosity, both of which were the results of lipid metabolism inability.

### Different components of SNF1 pathway make unequal contributions to the pathogenicity

**Figure 8:**
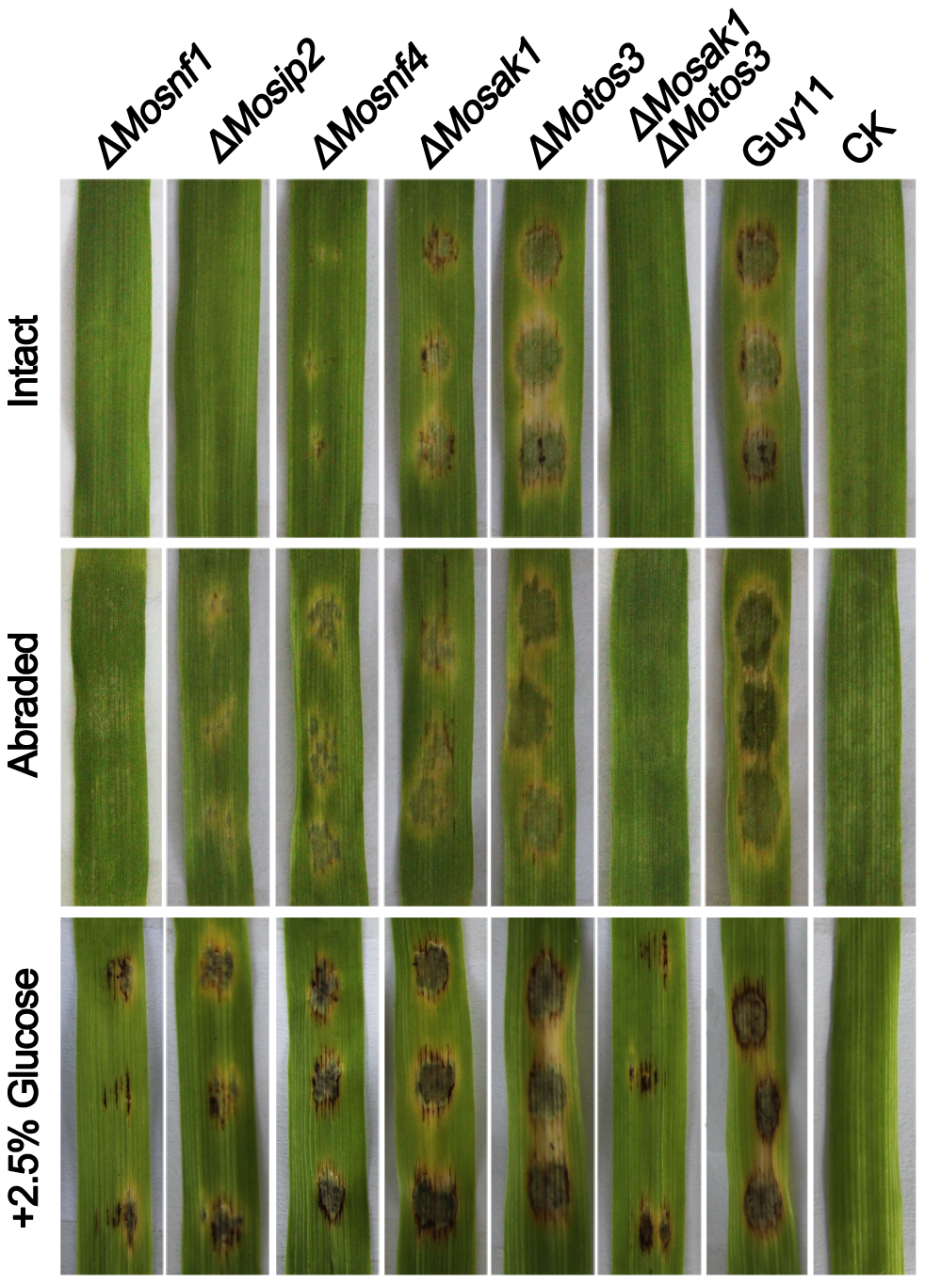
Pathogenicity assay on detached barley leaves. Intact and abraded barley leaves were inoculated with 20 µl conidial suspensions (1×10^5^ conidia/ml) of the tested strains for 4 days before photography. *ΔMosnf1*, *ΔMosip2*, *ΔMosnf4*, *ΔMosak1*, and *ΔMosak1ΔMotos3* were deficient in appressorium-mediated infection of intact barley leaves. Elevated virulence was observed in *ΔMosip2*, *ΔMosnf4*, and *ΔMosak1* when tested on abraded barley leaves, while *ΔMosnf1* and *ΔMosak1ΔMoto3* were still non-pathogenic. Addition of 2.5% glucose to conidial suspensions could evidently, yet partially, restored the virulence of the defective mutants.

**Figure 9:**
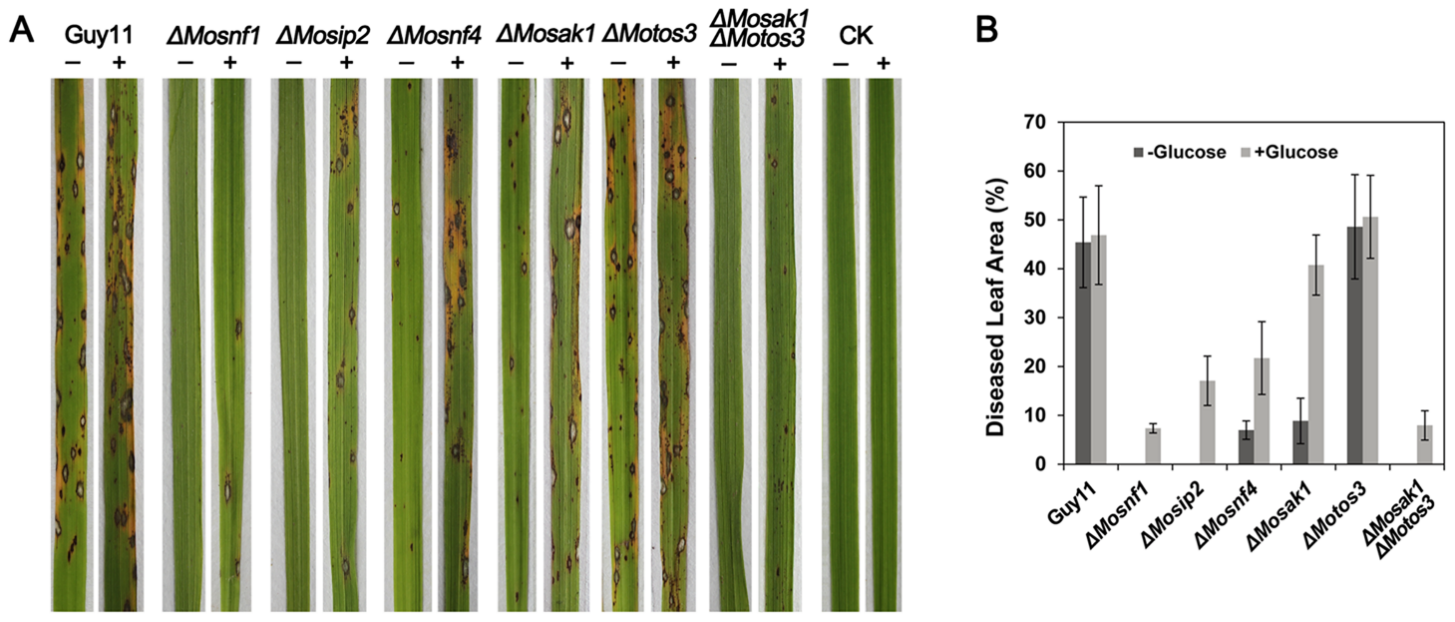
Spray inoculation assay with rice seedlings. Conidial suspensions (1×10^5^ conidia/ml) with (+) or without (-) 2.5% glucose were evenly sprayed onto rice leaves for 7 days before photographing the typical infected leaves (**A**) and calculating the percentage of diseased leaf area (**B**).

Due to the significant influence on turgor genesis, the pathogenicity of *ΔMosnf1* was severely impaired (Figure 8 and Figure 9), consistent with findings from previous study [23]. However, the exact reason why *ΔMosnf1* lost virulence has not been determined. Besides, other proteins of SNF1 pathway do not perform equal functions as MoSnf1 does in various processes, it is therefore necessary to investigate whether they contribute differently to plant infection by *M. oryzae*.

Conidial suspensions, freshly collected from Guy11 and the mutants, were appropriately diluted and set to infect host leaves. Similar to *ΔMosnf1*, both *ΔMosip2* and *ΔMosak1ΔMotos3* failed to elicit any visible disease lesions on barley and rice leaves (Figure 8 and Figure 9), while the virulence of *ΔMosnf4* and *ΔMosak1* was sharply attenuated, causing only tiny and restricted lesions on rice leaves (7.0% and 8.9% diseased leaf area, respectively, vs. 45.4% in Guy11) (Figure 8 and Figure 9). In contrast, inoculation with spores from Guy11, *ΔMotos3*, and the complemented strains resulted in the development of typical rice blast symptoms (Figure 8, Figure 9, and Figure S4). Subsequently, barley leaves were abraded to examine the invasive growth of the mutants *in planta*. Interestingly, *ΔMosip2*, *ΔMosnf4*, and *ΔMosak1* all developed extensive lesions although with compromised spread rate compared to Guy11 and *ΔMotos3* (Figure 8). However, the wounded leaf areas underneath conidial droplets of *ΔMosnf1* and *ΔMosak1ΔMotos3* remained healthy, suggesting the two mutants also lost the ability to proliferate within host tissues (Figure 8).

**Figure 10:**
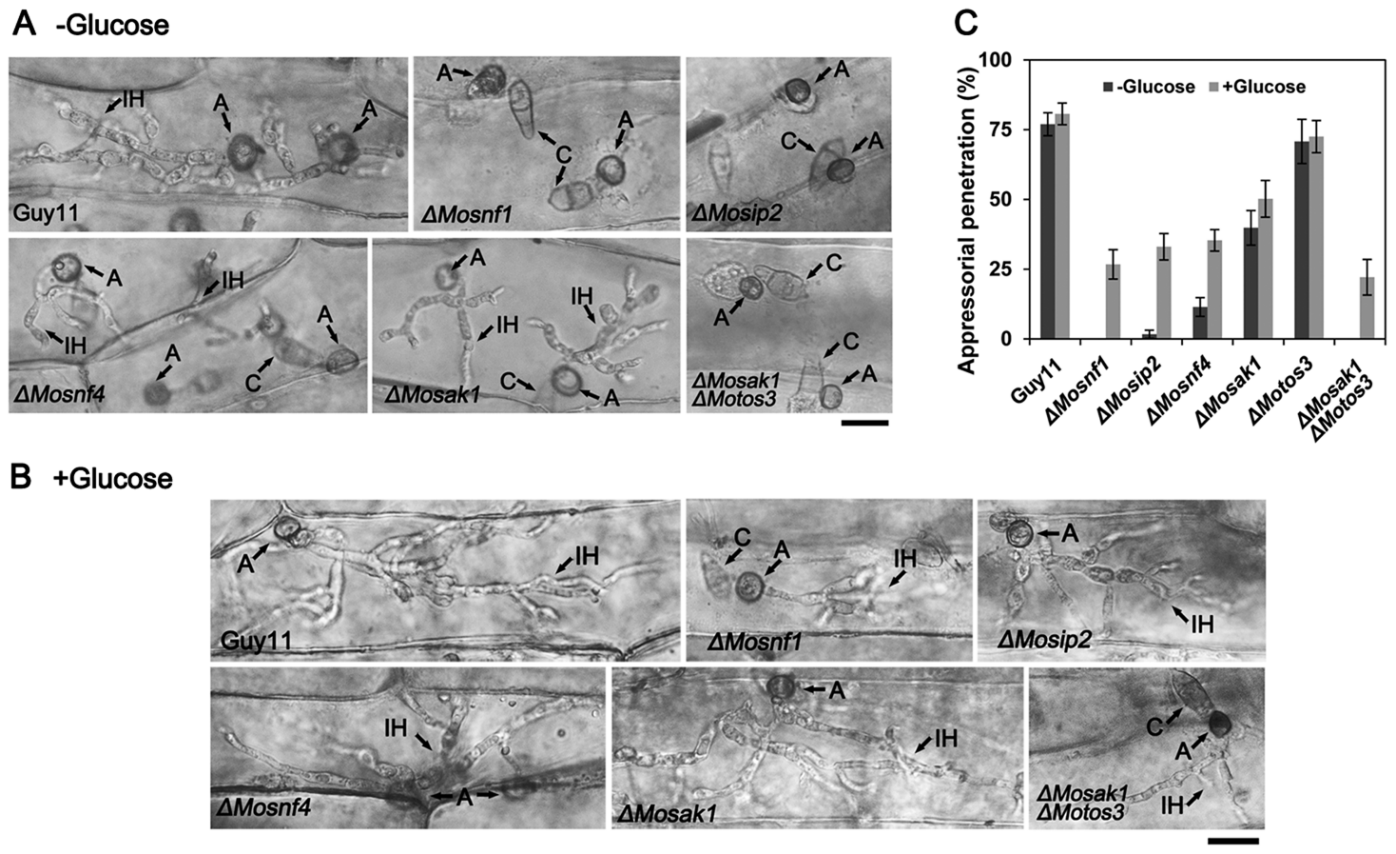
Penetration assay on barley epidermal cells. Barley leaves were inoculated with conidial suspensions supplemented without (**A**) or with (**B**) 2.5% glucose for 48 h, and then decolored by methanol before observation. A, appressorium; C, conidium; IH, invasive hyphae. Bars  = 15 µm. (**C**) Percentage of appressoria capable of penetration was counted at 48 hpi, and the results were presented as means and standard deviations.

To further test the appressorial function, penetration and invasive growth were microscopically observed after inoculating barley leaves with conidial suspensions from WT and the mutants. Consistent with the pathogenicity assay, the penetration rates of *ΔMosnf4* and *ΔMosak1* (11.4% and 39.8%, respectively) were strongly compromised when compared to Guy11 (77.0%) at 48 hpi (Figure 10C), although some mutant appressoria developed aggressive invasive hyphae (Figure 10A). The rate of appressoria forming infectious hyphae in *ΔMotos3* (70.8%) was comparable to WT (Figure 10C). Whereas *ΔMosnf1*, *ΔMosak1ΔMotos3*, and the majority of *ΔMosip2* (98.3%) appressoria were incapable of penetrating barley epidermal cells at 48 hpi (Figure 10A and 10C), revealing that their reduced virulence was the result of perturbed appressorial function.

The above observations indicate the different components of SNF1 pathway play unequal roles in pathogenicity-related processes. Although the phenotypic defects of *ΔMosak1* were much more severe than *ΔMotos3*, additional deletion of *MoTOS3* led to a similar phenotype as what occurred in *ΔMosnf1*, suggesting that MoSak1 together with MoTos3 make contributions to MoSnf1 activation.

### External carbon supplement relieved the defects of mutants with impaired SNF1 function

Since the infection of *M. oryzae* occurs in a nutrient-free environment, the development of appressoria and subsequent infectious hyphae must be nutritionally supported by the degradation of conidial reserves [31]. Previous studies have proved the generation of acetyl-CoA via fatty acid β-oxidation is a prerequisite for appressorium-mediated plant infection, as acetyl-CoA is the substrate to synthesize glucans and chitin, which are required for cell wall biosynthesis [31], [35]. Given the decreased amount of peroxisomes during appressorial development, we investigated whether the mutant defects in appressorial function and invasive growth could be rescued by external carbon source, which can supply acetyl-CoA and other intermediates via the glycolytic pathway and citric acid cycle independent of peroxisomal fatty acid β-oxidation.

As expected, pathogenic defects of all the mutants were partially complemented in the presence of exogenous glucose (Figure 8 and Figure 9). In order to explore the exact reason, we monitored the changes in appressorial structure and function in detail. When supplemented with 2.5% glucose, the SNF1 pathway mutants were restored to a large extent in terms of appressorial size (Figure 3B) and cell wall porosity (the ratio of plasmolysis to cytorrhysis in 1.7 M glycerol  = 0.24±0.05 and 0.26±0.07 in *ΔMosnf1* and *ΔMosak1ΔMotos3*, respectively, dramatically lower than the data listed in Figure 7B). The appressorial penetration ability was also raised significantly in *ΔMosnf1* (0 to 26.7%), *ΔMosip2* (1.69% to 33.1%), *ΔMosnf4* (11.4% to 35.3%), *ΔMosak1* (39.8% to 50.2%), and *ΔMosak1ΔMotos3* (0 to 22.1%), compared to the slight improvement in Guy11 (77.0% to 80.7%) and *ΔMotos3* (70.8% to 72.5%) at 48 hpi (Figure 10C). Furthermore, exogenous glucose enabled *ΔMosnf1*, *ΔMosip2*, and *ΔMosak1ΔMotos3* to develop branched secondary hyphae, in striking contrast to the almost inhibited penetration observed in the absence of nutrient (Figure 10A and 10B).

These results indicate that external glucose metabolism could partially compensate the insufficient peroxisomal function and rescue the phenotypic defects of SNF1 pathway mutants, which is consistent with the effect of extra glucose on *Δpex6* mutants [35], [44].

## Discussion

As a hemibiotrophic pathogen, *M. oryzae* has to regulate its cellular metabolic activities to adapt to nutrient unavailability during the early infection stage [24]. In yeast, the SNF1 signaling pathway plays a central role in regulating energy status by its involvement in carbon catabolite derepression, a mechanism to ensure the utilization of unfavorable carbon sources when glucose is deprived [3]. Previous studies on SNF1 function in phytopathogenic fungi have uncovered its great influences on the expression of cell wall degrading enzymes (CWDEs) and the utilization of alternative sugars, both of which are subject to carbon catabolite repression (CCR) [16], [17], [18], [19]. However, the *M. oryzae* MoSnf1 protein was reported not to preserve such regulatory role [23]. Recently, trehalose-6-phosphate synthase 1 (Tps1) was recognized as a glucose-6-phosphate sensor, which cooperates with its downstream inhibitors Nmr1-3 to mediate CCR regulatory system in *M. oryzae* [45]. Besides, the expression pattern of CWDEs was found to be disturbed in *MoTPS1* disruption mutant [45]. These reports suggest the components of metabolic regulatory systems have changed greatly in *M. oryzae*, likely not involving MoSnf1 [46]. This study set out to investigate how MoSnf1 acts as a virulence determinant and delve into the function of SNF1 pathway in *M. oryzae*.

In fungi, where non-fermentable compounds like fatty acids and acetate can serve as sole source of carbon and energy, the acetyl-CoA must be converted to C4 compounds via the glyoxylate cycle, allowing gluconeogenesis [5], [47]. Peroxisome plays an essential role in this process, as it could serve as the location where fatty acid beta-oxidation occurs to generate acetyl-CoA [48], meanwhile many glyoxylate cycle enzymes are also peroxisomal [49]. In *M. oryzae* [30], [33], [34], [41], *Colletotrichum lagenarium* [44], and *Fusarium graminearum* [50], mutants with aberrant peroxisome function showed severe defects in utilization of lipids, fatty acids, and acetate. Yeast *Δsnf1* is devoid of peroxisomal structures and fails to survive on media with non-fermentable carbon sources [11], [38]. However, we found *ΔMosnf1* possessed abnormal other than abolished peroxisomes with enlarged size in the mycelia. The poor growth of *ΔMosnf1* on fatty acids or NaAC-contained media suggested an aberrant function of the enlarged peroxisomes therein. In *S. cerevisiae*, *PEX1/PEX11*, *FOX2*, and *ICL1*, all of which are regulated by Snf1 [11], play an important role in peroxisomal biosynthesis and proliferation [51], peroxisomal fatty acid beta-oxidation [48], and the glyoxylate cycle [52], respectively. However, gene expression profiling by qRT-PCR revealed that *M. oryzae* homologs, *MoPEX1* (MGG_09299), *MoPEX11* (MGG_08896), *MoFOX2*/*MoMFP1* (MGG_06148), and *MoICL1* (MGG_04895), were up-regulated when induced by olive oil or triolein in both WT and *ΔMosnf1*, while no significant differences in the up-regulation folds were observed (Figure S5). Thus, the regulatory mechanism controlling peroxisome function by MoSnf1 remains obscure, which may involve the derepression of other key factors not tested in this study.

Consistent with previous studies [53], peroxisomal targeting signals of WT and *ΔMotos3* were distributed as cytoplasmic punctate spots, but not visible in the vacuoles of germinating conidia until over 6 hpi. However, accelerated degradation of PTS1 signals was observed in the ungerminated conidia of SNF1 function-disturbed mutants. Unlike *ΔMopex* mutants with dispersed GFP-PTS1 localization in cytoplasm [30], [34], [35], punctate fluorescent spots were still present in *ΔMosnf1*, suggesting it retained the integral PTS1 import pathway and peroxisomal formation ability but failed to maintain adequate peroxisomes. One reason we deduced why *ΔMosnf1* performed the abnormal GFP-PTS1 sequential localization could be caused by the premature pexophagy. Another likelihood might be that *ΔMosnf1* was incapable to form adequate peroxisomes as Guy11 did, and the surplus GFP-PTS1 had to be delivered to vacuolar lumen for degradation directly.

Insufficient amount of peroxisomes could give rise to serious consequence in *M. oryzae*. It is known that enormous turgor pressure is required for the pathogen to physically penetrate the host surface, and its genesis relies on the substantial glycerol accumulation via rapid lipolysis during appressorial maturation [26]. In order to maintain the efficient lipid mobilization, fatty acids resulting from lipolysis demand to be transported to peroxisomes where they are metabolized via β-oxidation to form acetyl-CoA [29], [31], [35]. The resulting acetyl-CoA is not only the precursor in melanin synthesis but also supplies substrates to synthesize chitin and glucans via the glyoxylate shunt and gluconeogenesis [30], [32]. So peroxisome, the major β-oxidation location and acetyl-CoA sink, plays a central role in appressorial morphogenesis and function [37]. Mutants with dysfunctional peroxisomes display significant defects in lipid metabolism, melanin layer formation, appressorial wall porosity, turgor generation, and ultimately pathogenicity [30], [33], [34], [44]. Possibly due to the sharp decline of peroxisomal number, *ΔMosnf1* performed a significant retard in lipid droplets mobilization, and formed larger appressorial wall pore, coupled with the failure to generate enormous turgor and the loss of virulence. However, a distinct melanin layer, without significant differences in thickness, was observed between cell wall and plasma membrane in both *ΔMosnf1* and Guy11 appressorium, indicating that the defect in porosity of appressorial wall seemed not to be related to melanin biosynthesis. Consistent with *M. oryzae* mutants disturbed in peroxisomal function [30], [34], *ΔMosnf1* partially restored the appressorial morphology and pathogenicity when supplemented with external carbon source, which could compensate acetyl-CoA via the glycolytic pathway and citric acid cycle independent of peroxisomal function. Further quantification of diseased leaf area and penetration rate on hosts revealed that the restoration by external glucose was incomplete in *ΔMosnf1*. Thus, except for peroxisomal maintenance, MoSnf1 may be involved in other cellular processes required for virulence.

Apart from elucidating how MoSnf1 was involved in pathogenesis, we additionally elaborated the SNF1 pathway cascade in *M. oryzae* through targeted gene deletion strategy. The solo β and γ subunits of SNF1 complex, MoSip2 and MoSnf4 respectively, were identified and further confirmed based on the phenotypic evidences and their strong interaction with MoSnf1, as well as each other. The mutants without β or γ subunit exhibited Snf1^-^-like phenotype, but with weaker phenotypic defects compared to *ΔMosnf1*, indicating that MoSnf1 plays a major role in the kinase complex, while the complex integrity promotes MoSnf1 function conversely. In accordance with our findings, truncated yeast Snf1 kinase domain (residues 1–309), *snf1* (1–309), does not interact with the β or γ subunit, but has partial Snf1 function [54]. These results suggest the Snf1 kinase activity might occur by a mechanism independent of the β and γ subunits, although in a limited level. Besides, we additionally characterized two putative upstream Snf1-activating kinases, MoSak1 and MoTos3. However, the two kinase mutants acquired unique properties. *ΔMosak1* was defective in conidiogenesis, while the sporulation ability of *ΔMotos3* was three times as high as WT, suggesting an antagonistic effect exists between them. On the other hand, only removal of them both resulted in completely eliminated virulence, indicative of a collaborative relationship between the two kinases. The phenotypic changes in *ΔMosak1ΔMotos3* were comparable to those of *ΔMosnf1* in most aspects, but differences still existed such as shorter aerial hyphae and conidiophores, and reduced mycelial growth rate, implying the two kinases may regulate more processes apart from the SNF1 pathway. In *S. cerevisiae*, for example, the three upstream kinases, Sak1, Elm1, and Tos3, are also found to participate in the regulation of G protein signaling [55]. Furthermore, the sensitivity to Ca^2+^ in *ΔMosak1ΔMotos3* was comparable to that in *ΔMosak1*, but slighter than the SNF1 complex mutants (Figure S6), while the growth rate of *ΔMotos3* on calcium supplemented medium was similar to Guy11 (Figure S6). Therefore, there might be another kinase together with MoSak1 to regulate SNF1 activity under the certain stress. In yeast, although partially redundant function exists within the three Snf1-activating kinases, there is a clear preference to Sak1 in Snf1 activation [6], [7]. Likewise, the significant defects in *ΔMosak1* suggested MoSak1 occupies a predominant position in MoSnf1 regulation in *M. oryzae*, while MoTos3 plays an auxiliary role to our knowledge. The conclusion was further supported by the transcription pattern of *MoSAK1*, with a relatively higher expression increase than *MoTOS3* during infection-related processes.

Taken together, the integral SNF1 complex is crucial for various development patterns in *M. oryzae*, simultaneously the two upstream kinases are of great importance to SNF1 activity with the predominant status of MoSak1. We also demonstrate the SNF1 pathway retains the conserved role to enable the fungus to adapt to nutrient-free environment via its participation in peroxisomal maintenance and lipid metabolism, thus acting as an important pathogenicity-related module. However, the exact mechanism by which SNF1 pathway interplays with peroxisomal dynamics remains elusive in *M. oryzae*. This study provides some insights for further research on the conservation and divergence of SNF1 pathway among fungi.

## Materials and Methods

### Strains and culture conditions

*M. oryzae* wild-type strain Guy11 and all the derivative transformants were grown routinely on complete medium (CM) at 25°C with a 16 h fluorescent light photophase [56]. Growth phenotypic comparisons of Guy11 and the mutant strains were performed on MM supplemented with glucose-substituted non-fermentable carbon substrates (1% Tween 80, 1% olive oil, 1% triolein, or 50 mM sodium acetate) for 10 d, or on CM in addition with 0.3 M Calcium chloride for 7 d.

### Quantitative RT-PCR analysis

Fungal tissues used for qRT-PCR assay included fresh mycelia cultured in liquid CM for 3 days, conidia harvested from 10-day-old CM cultures, appressoria incubated on hydrophobic surfaces 8 hours postincubation (hpi), and infected barley leaves collected at 72 hpi. For lipid induced transcription analysis, strains were first incubated in liquid CM for 48 h, and then transferred to liquid MM-C supplemented with 1% glucose, 1% triolein, or 1% olive oil to induce 6 h. The extraction of total RNAs from above samples followed a previously described protocol [56] with the Trizol reagent (Takara). First-strand cDNA was synthesized from 800 ng total RNA according to SYBR ExScriptTM RT-PCR kit (Takara). Quantitative real-time PCR was performed as previously described [56] with SYBR Premix ExTaq (Takara) on a Mastercycler ep realplex thermo cycler (Eppendorf). Primers used for qRT-PCR assays were listed in Table S2. The relative expression level of each gene was calculated by the 2^-ΔΔCt^ method [57] with β-tubulin (MGG_00604) as reference. Data were collected from three independent experiments with three replicates, and a representative set of results was displayed.

### Generation of gene knockout mutants and complementation

The gene deletion vectors were constructed based on double-joint PCR strategy [58]. Take the construction of *MoSIP2* knockout vector for example: approximate 1.1 kb up- and down-stream regions of *MoSIP2* locus were amplified from *M. oryzae* genomic DNA using primers SIP2up-1/2 and SIP2dn-1/2. A 1.4 kb *hph* cassette was cloned from pCB1003 with primers HPH-1/2. The three fragments were joined together in the second round PCR, the product of which acted as the template for the final amplification with nest primers SIP2-N1/2. The 3.4 kb double-joint PCR product which contained the flanking sequences and *hph* cassette was inserted into the XhoI/XbaI sites of pCAMBIA1300 to obtain the targeted gene deletion vector. Using a similar construction strategy, targeted gene deletion vectors of *MoSNF1*, *MoSNF4*, *MoSAK1*, and *MoTOS3* were generated. These vectors were introduced into *M. oryzae* WT strain via *Agrobacterium tumefaciens*-mediated transformation (ATMT) [59]. To generate the double mutant, the *MoTOS3* deletion vector carrying sulfonylurea resistance allele of *Magnaporthe ILV1* gene (amplified with primes SUR-1/2) was introduced into *ΔMosak1* background. Gene deletion events were screened by PCR and further confirmed by Southern blot analysis. The complementary fragments, which contained the entire targeted genes and their native promoter and terminator regions, were amplified by PCR with primers C1/2 (Table S2) and inserted into a modified pCAMBIA1300 vector, which contained a geneticin resistance gene. The resultant constructs were randomly inserted into the genome of the corresponding mutants via ATMT. The complemented transformants were screened on the selective media containing 800 µg/ml geneticin. As the complementation of *MoSNF1* had been carried out before [23], we didn't repeat the work here. Southern blot analysis was performed according to the digoxigenin (DIG) high prime DNA labeling and detection starter Kit I (Roche). Primer pairs used in DNA manipulation events were listed in Table S2.

### Yeast two-hybrid assay

The Yeast two-hybrid assay was carried out according to the BD Matchmaker Library Construction & Screening Kits instruction (Clontech). The coding sequence of each candidate gene was amplified with primer pairs listed in Table S2, each of which was incorporated with 15 bases of homology with the ends of the linearized vector. The construct strategy was according to the In-Fusion HD Cloning Kit (Clontech). Consequently, the bait vector pGBKT7-MoSnf1 and each of the prey vector pGADT7-MoSip2, MoSnf4, MoSak1, MoTos3, were co-transformed into yeast strain AH109. The positive tranformants on SD-Leu-Trp medium were further tested on SD-Ade-His-Leu-Trp medium. In order to test the interaction between MoSip2 and MoSnf4, the bait vector pGBKT7-MoSip2 was constructed and co-transformed with pGADT7-MoSnf4 into AH109 as described above. The positive and negative control strains used in the assay were from above mentioned Kit.

### Assays for sporulation, appressorium formation, and plant infection

Quantitative measurement of conidiation was assayed with 10-day-old cultures grown on CM plates [23], while the aerial hyphal and conidial development was monitored as described previously [56], [60]. To allow appressorium formation, 40 µl of conidial suspensions adjusted to 5×10^4^ conidia/ml were placed on plastic cover slips (Fisher) under humid conditions at room temperature. The conidial germination and appressorium formation were observed under a light microscope after 6 h, 12 h, and 24 h postincubation. To monitor appressorium-mediated penetration and invasive hyphae growth, leaf explants of barley (*Hordeum vulgare* cv. ZJ-8) were inoculated with 20 µl of the above conidial suspensions and treated according to previously described protocols [61] at 48 hpi before observation.

For rice infection assay, conidial suspensions were diluted to 1×10^5^ conidia/ml in 0.2% gelatin, and 4 ml of each suspension was sprayed onto the 3–4 leaf stage rice seedlings (*Oryza sativa* cv.CO39). Diseased leaves were examined and imaged at 7 d after inoculation. For further disease severity assessment, the diseased leaf area (%) was quantified by calculating the pixels under lesion and healthy areas of diseased leaf blades using the Histogram command of Photoshop CS5 [62]. For barley infection assay, 20 µl 1×10^5^ conidia/ml suspensions were deposited on leaf segments of 7-day-old seedlings of barley ZJ-8 incubated in a humid chamber at 25°C for 4 d. For wounded leaf inoculation, the barley leaf surfaces were slightly abraded to remove the cuticle before infection assay. Each test was repeated three times.

### Staining methods and microscopy

Eight-day-old conidia were used in the cytological studies. Conidia suspensions diluted to 1×10^5^/ml were incubated on hydrophobic films to form appressoria in a humid chamber at 25°C. To stain vacuolar lumen, CMAC (7-amino-4-chloromethylcoumarin) was used as previously described [63]. Observation of lipid bodies during appressorial development was carried out at 4 h, 12 h, 24 h, 48 h, and 96 h postincubation with Nile Red staining as previously described [28]. Because the melanin layer of the appressorium might interfere with the lipid droplets visualization, samples were treated with 10 µg/ml tricyclazole, a melanin biosynthesis inhibitor, before induced. To investigate the localization of PTS1-containing proteins, the GFP-PTS1 fusion vector p1300NMGFPA (kindly provided by Dr. Jiaoyu Wang), which carried geneticin resistance and was under control of *MPG1* promoter, was introduced into Guy11, *ΔMosnf1*, *ΔMosip2*, *ΔMosnf4*, *ΔMosak1*, *ΔMotos3*, and *ΔMosak1ΔMotos3*, respectively via ATMT. For quantitative analysis of lipid droplets mobilization and PTS1 localization based on fluorescence, more than 100 conidia and appressoria were analyzed for each strain in triplicate. The light and epifluorescence microscopic examination was conducted under an Eclipse 80i microscope (Nikon) equipped with a Plan APO VC 100X/1.40 oil objective. To visualize the GFP-PTS1 signals and CMAC-stained vacuoles in detail, ZEISS LSM780 inverted confocal microscope (Carl Zeiss Inc.) equipped with a 30 mW Argon laser was used. To detect the melanin layer of the appressorium under a JEM-1230 electron microscope (JEOL, Tokyo, Japan) operating at 70 kV, conidia collected from WT and mutants were induced on barley leaves, and the 48 h postincubation segments were treated as described in [43].

### Measurement of appressorial turgor and cell wall porosity

Forty microlitre of conidial suspensions with a density of 1×10^5^ conidia/ml were dropped on plastic cover slips (Fisher) and incubated in a moist chamber for 48 h. Appressorial turgor was estimated by performing incipient cytorrhysis (cell collapse) assays as described previously [43]. The porosity of an appressorial wall was evaluated by plasmolysis/cytorrhysis assay as described previously [64], where polyethylene glycols (PEGs) of different average molecular weights were added to 48 h appressoria to generate an external osmotic pressure of 4 MPa. Each assay was repeated three times.

## Supporting Information

Figure S1 **Targeted gene replacements of** ***MoSNF1*** **,** ***MoSIP2*** **,** ***MoSNF4*** **,** ***MoSAK1*** **, and** ***MoTOS3*** **.** (**A**) Targeted gene deletion of *MoSAK1*. The gene deletion vector was constructed based on double joint PCR. The orientations and positions of primers SAK1up-1/2, SAK1dn-1/2, HPH-1/2, and SAK1-N1/2 are indicated as 1–8, respectively, with small arrows. And the deletion event was verified by Southern blot analysis. *Xho*I-digested genomic DNAs were hybridized with the 1.1 kb probe amplified with primes SAK1pb-1/2. As expected, a single band was shifted from WT 2.6 kb to 4.6 kb in *ΔMosak1* and *ΔMosak1ΔMotos3*. The complemented transformant with a single-copy epic insertion was confirmed by two distinct bands observed. The targeted gene replacements of *MoTOS3* (**B**), *MoSNF1* (**C**), *MoSIP2* (**D**), and *MoSNF4* (**E**) were carried out by the similar strategy.
(TIF)

Figure S2 **Subcellular localization of GFP-PTS1 in Guy11 and** ***ΔMosnf1*** **during appressorial development.** Conidia of Guy11 and *ΔMosnf1* were incubated on the surfaces of hydrophobic films and observed at the indicated time points. Contrast to the punctate peroxisomes in the nascent appressoria of WT, GFP-PTS1 was almost absent in the *ΔMosnf1* appressoria. Arrows denote fluorescence-contained vacuoles of WT conidia. Bars  = 5 µm.
(TIF)

Figure S3 **Ultrastructural analysis of the appressorium cell wall.** Appressoria were allowed to form on barley leaves for 48 h, and the ultrathin sections were processed for transmission electron microscopy. The melanin layers (indicated by the arrows) were detected in the wild type and mutant strains. Bar  = 0.5 µm.
(TIF)

Figure S4 **Recovery of pathogenicity in the complemented strains.** (**A**) Pathogenicity assay on detached barley leaves. Diseased leaves were photographed at 4 dpi. (**B**) Spray inoculation assay on rice leaves. Diseased leaves were photographed at 7 dpi.
(TIF)

Figure S5 **Expression profiles of** ***MoPEX1*** **,** ***MoPEX11*** **,** ***MoMFP1*** **, and** ***MoICL1*** **in the wild type (A) and** ***ΔMosnf1*** **(B) strains after induced in fatty acid media for 6 h.**
(TIF)

Figure S6 **Effect of calcium excess on the growth rate of SNF1 pathway mutants.** CM agar plates added with 0.3 M CaCl_2_ were used to culture strains for 7 days. Different degrees of sensitivity existed between the SNF1 complex mutants and the upstream kinase mutants.
(TIF)

Table S1 **Characteristics of SNF1 complex subunits and the upstream Snf1-activating kinases in** ***M. oryzae*** **.**
(DOC)

Table S2 **List of primers used in this study.**
(DOCX)
