# Supplementary figures and images for: Crosstalk between SNF1 Pathway and the Peroxisome-Mediated Lipid Metabolism in Magnaporthe oryzae
Source: PLoS One. 2014 Aug 4;9(8):e103124. doi: 10.1371/journal.pone.0103124 (PMC4121083; doi:10.1371/journal.pone.0103124)

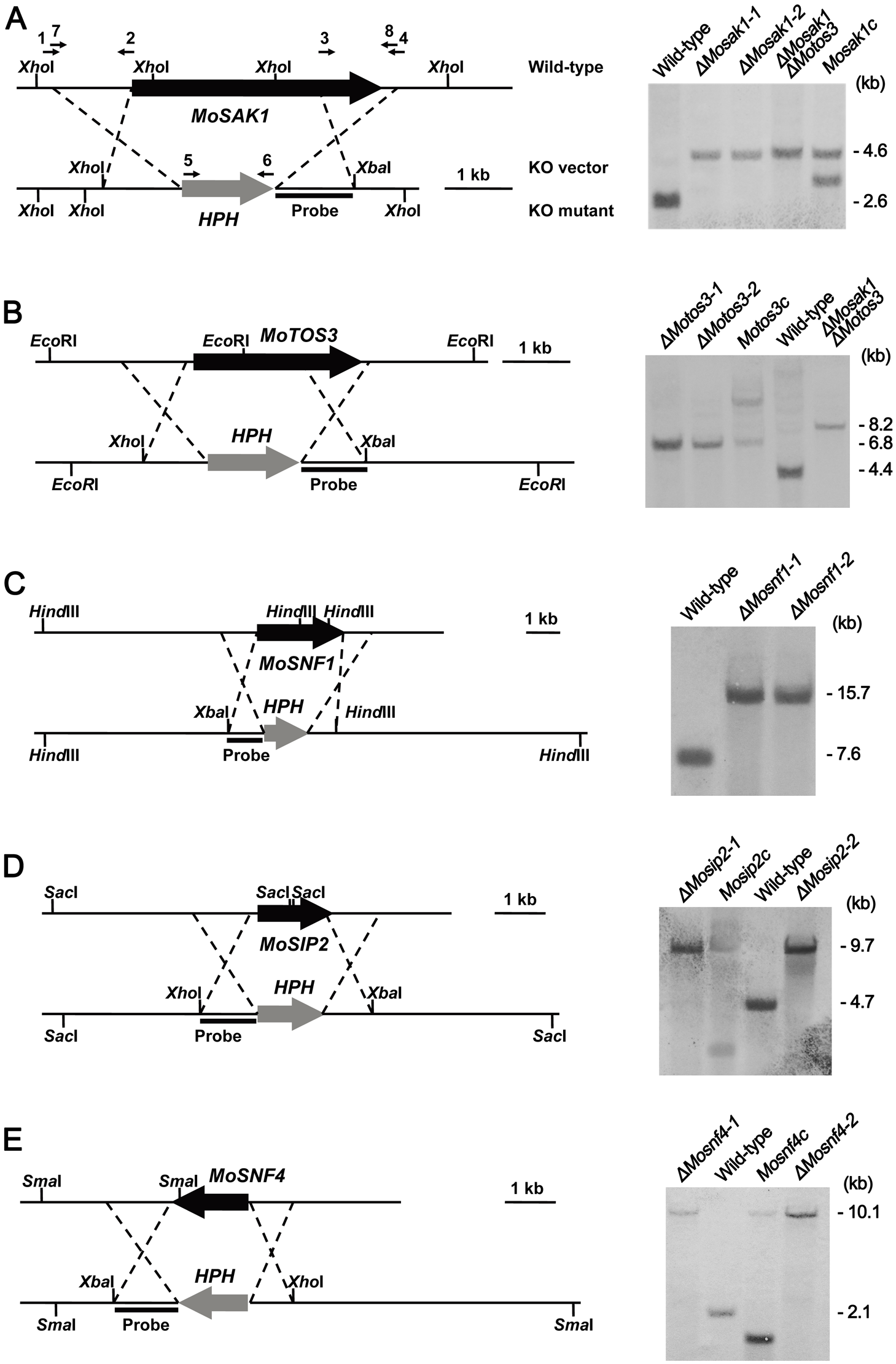

Supplement: Figure S1 — Targeted gene replacements of MoSNF1 , MoSIP2 , MoSNF4 , MoSAK1 , and MoTOS3 . (A) Targeted gene deletion of MoSAK1. The gene deletion vector was constructed based on double joint PCR. The orientations and positions of primers SAK1up-1/2, SAK1dn-1/2, HPH-1/2, and SAK1-N1/2 are indicated as 1–8, respectively, with small arrows. And the deletion event was verified by Southern blot analysis. XhoI-digested genomic DNAs were hybridized with the 1.1 kb probe amplified with primes SAK1pb-1/2. As expected, a single band was shifted from WT 2.6 kb to 4.6 kb in ΔMosak1 and ΔMosak1ΔMotos3. The complemented transformant with a single-copy epic insertion was confirmed by two distinct bands observed. The targeted gene replacements of MoTOS3 (B), MoSNF1 (C), MoSIP2 (D), and MoSNF4 (E) were carried out by the similar strategy. (TIF) [file pone.0103124.s001.tif]

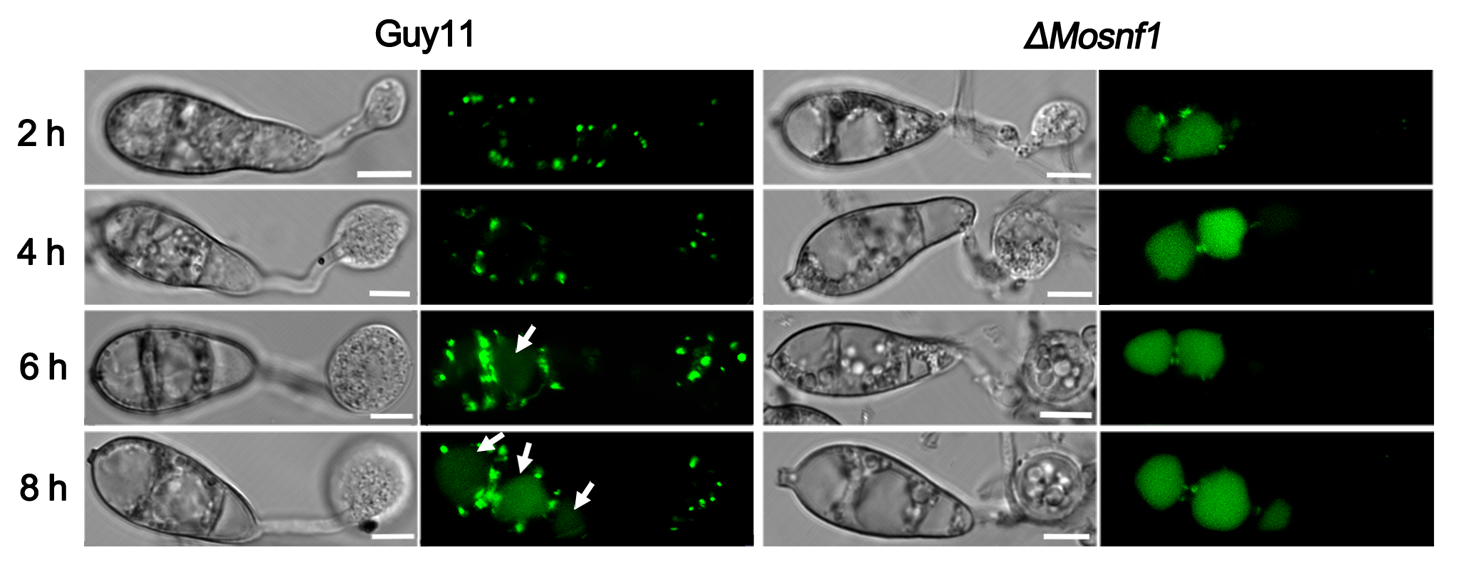

Supplement: Figure S2 — Subcellular localization of GFP-PTS1 in Guy11 and ΔMosnf1 during appressorial development. Conidia of Guy11 and ΔMosnf1 were incubated on the surfaces of hydrophobic films and observed at the indicated time points. Contrast to the punctate peroxisomes in the nascent appressoria of WT, GFP-PTS1 was almost absent in the ΔMosnf1 appressoria. Arrows denote fluorescence-contained vacuoles of WT conidia. Bars = 5 µm. (TIF) [file pone.0103124.s002.tif]

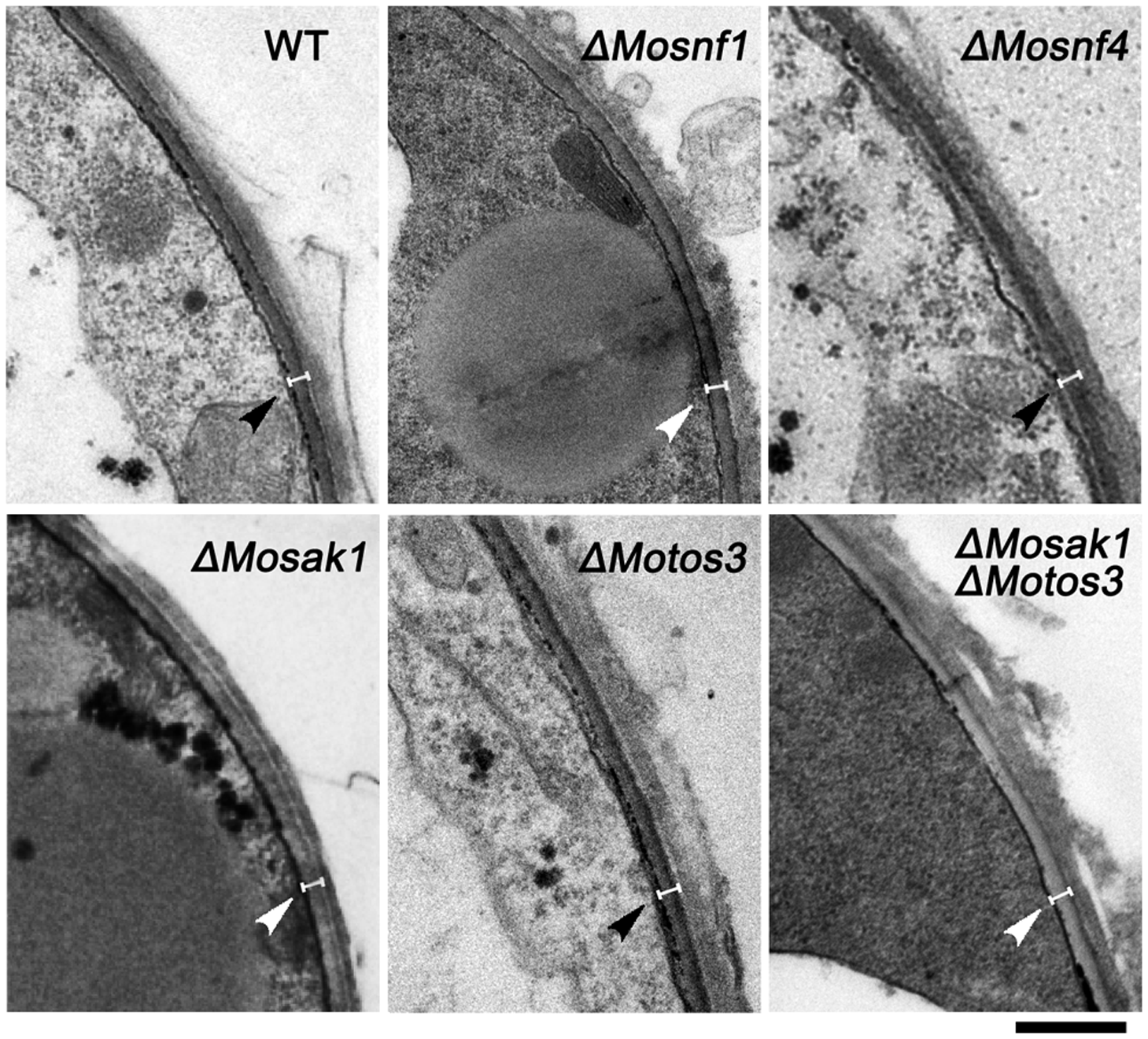

Supplement: Figure S3 — Ultrastructural analysis of the appressorium cell wall. Appressoria were allowed to form on barley leaves for 48 h, and the ultrathin sections were processed for transmission electron microscopy. The melanin layers (indicated by the arrows) were detected in the wild type and mutant strains. Bar = 0.5 µm. (TIF) [file pone.0103124.s003.tif]

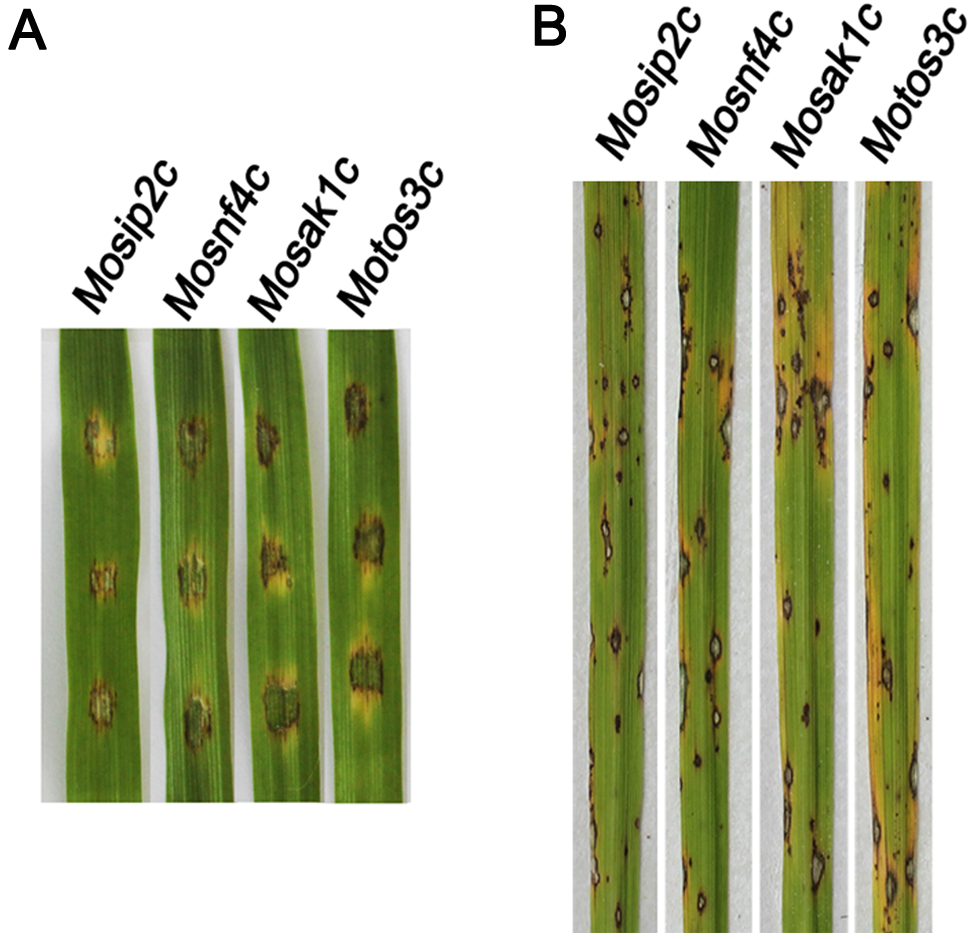

Supplement: Figure S4 — Recovery of pathogenicity in the complemented strains. (A) Pathogenicity assay on detached barley leaves. Diseased leaves were photographed at 4 dpi. (B) Spray inoculation assay on rice leaves. Diseased leaves were photographed at 7 dpi. (TIF) [file pone.0103124.s004.tif]

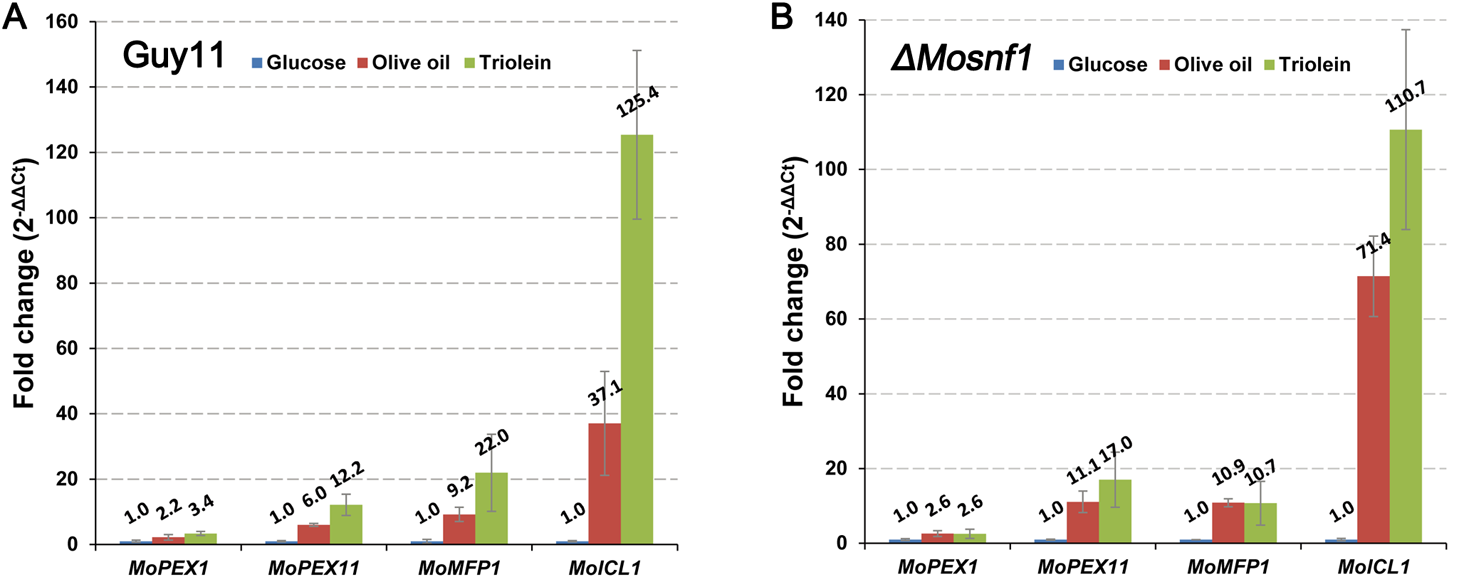

Supplement: Figure S5 — Expression profiles of MoPEX1 , MoPEX11 , MoMFP1 , and MoICL1 in the wild type (A) and ΔMosnf1 (B) strains after induced in fatty acid media for 6 h. (TIF) [file pone.0103124.s005.tif]

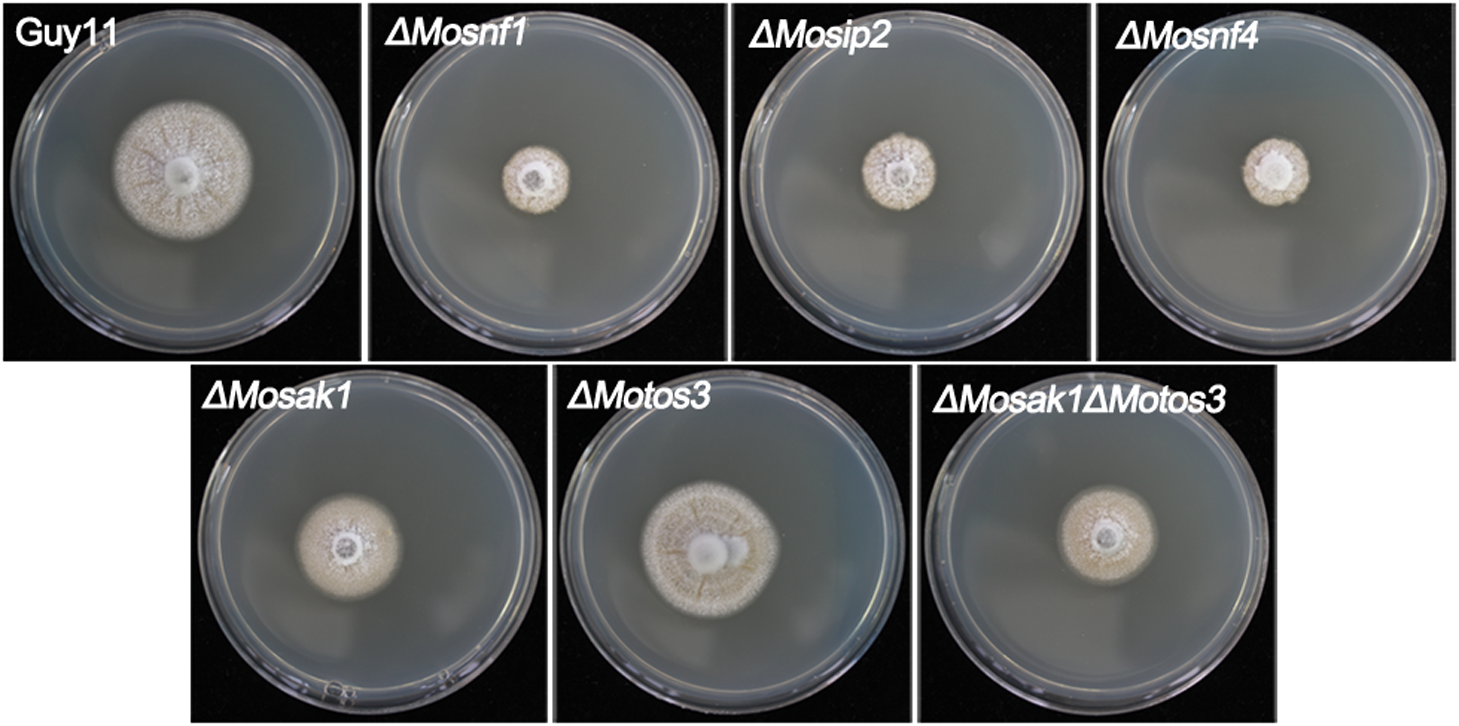

Supplement: Figure S6 — Effect of calcium excess on the growth rate of SNF1 pathway mutants. CM agar plates added with 0.3 M CaCl2 were used to culture strains for 7 days. Different degrees of sensitivity existed between the SNF1 complex mutants and the upstream kinase mutants. (TIF) [file pone.0103124.s006.tif]
