# Supplementary material for: Crosstalk between SNF1 Pathway and the Peroxisome-Mediated Lipid Metabolism in Magnaporthe oryzae
Source: PLoS One. 2014 Aug 4;9(8):e103124. doi: 10.1371/journal.pone.0103124 (PMC4121083; doi:10.1371/journal.pone.0103124)
Supplement: Table S1 — Characteristics of SNF1 complex subunits and the upstream Snf1-activating kinases in M. oryzae . (DOC) [file pone.0103124.s007.doc]

**Table S1.** Characteristics of SNF1 complex subunits and the upstream Snf1-activating kinases in *M. oryzae.*

| Function | *S. cerevisiae* gene | Best *M. oryzae* hit | *e*-value | Protein sequence analysis and classification |
| --- | --- | --- | --- | --- |
| α catalytic subunit | *SNF1* | MGG_00803 | 3e-170 | 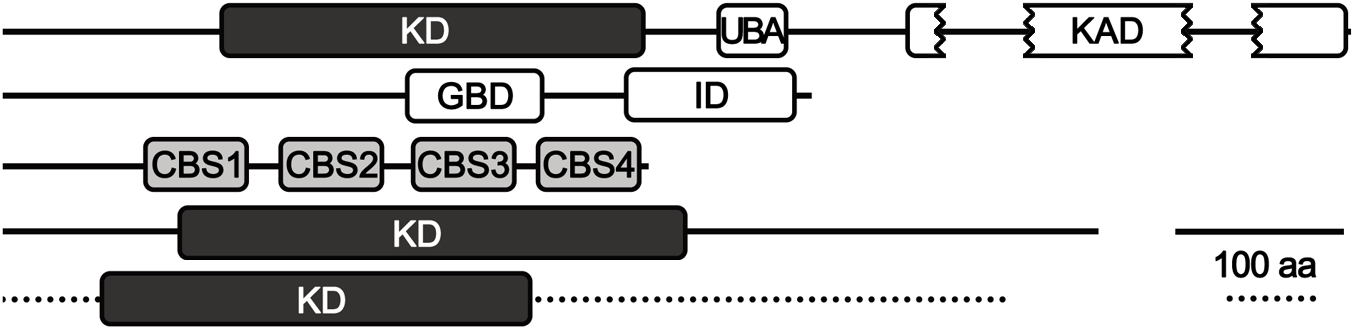 |
| β regulatory subunit | *SIP1/ SIP2/ GAL83* | MGG_06930 | 6e-07/2e-34/1e-32 |  |
| γ regulatory subunit | *SNF4* | MGG_04005 | 3e-122 |  |
| Upstream kinase | *TOS3/ SAK1/ ELM1* | MGG_06421 | 1e-51/4e-48/5e-32 |  |
|  |  | MGG_07003 | 2e-35/1e-39/2e-26 |  |

1. Hunter S, Jones P, Mitchell A, Apweiler R, Attwood TK, et al. (2012) InterPro in 2011: new developments in the family and domain prediction database. Nucleic Acids Res 40: D306-312.

KD, protein kinase domain (IPR000719); UBA, ubiquitin-associated domain (IPR013896); KAD, kinase associated domain 1 (IPR001772); GBD, carbohydrate or glycogen binding domain (IPR005102); ID, 5-AMP-activated protein kinase, beta subunit, interaction domain (IPR006828); CBS, CBS domain (IPR000644), pairs of these domains have been termed a Bateman domain.
