# Supplementary material for: Crosstalk between SNF1 Pathway and the Peroxisome-Mediated Lipid Metabolism in Magnaporthe oryzae
Source: PLoS One. 2014 Aug 4;9(8):e103124. doi: 10.1371/journal.pone.0103124 (PMC4121083; doi:10.1371/journal.pone.0103124)
Supplement: Table S2 — List of primers used in this study. (DOCX) [file pone.0103124.s008.docx]

**Table S2.** List of primers used in this study.

| **Name Sequence (5’ - 3’)** | |
| --- | --- |
| HPH-1 | TAGTGGAGGTCAACAATGAATG |
| HPH-2 | CATCTACTCTATTCCTTTGCCC |
| SNF1up-1 | GCGAGTAAGCGACTGTGAAA |
| SNF1up-2 | CATTCATTGTTGACCTCCACTAACAGCGACGGGAAACAAATAA |
| SNF1dn-1 | GGGCAAAGGAATAGAGTAGATGAGACCAGTCCATTCCCCTTCC |
| SNF1dn-2 | AGACCTCCTGTCGCAACCTA |
| SNF1-N1 | GGCTCTAGACAATCAAGGTCGGTTGGTAC |
| SNF1-N2 | CGAAGCTTTTGCTCTTTGAAGTCTCCCA |
| SNF1pb-1 | CAATCAAGGTCGGTTGGTAC |
| SNF1pb-2 | CAGCGACGGGAAACAAATAA |
| SIP2up-1 | TTCAGTGGAGGAACTGGTCA |
| SIP2up-2 | CATTCATTGTTGACCTCCACTAACCTATCGGCAAGGGTATGGT |
| SIP2dn-1 | GGGCAAAGGAATAGAGTAGATGATTGAACCACCTTGCCACTTC |
| SIP2dn-2 | CCTTCTCGGGTATCCTGACT |
| SIP2-N1 | GACTCGAGCATTAGCAAGCGATGACGAT |
| SIP2-N2 | CCTCTAGACATGTTGGTAGCACGCAGGT |
| SIP2-C1 | CGTCTAGATAGGCGAGACCGAGTCCATA |
| SIP2-C2 | TAGGATCCGACCGTCTTTGCCATGCTAG |
| SIP2pb-1 | CATTAGCAAGCGATGACGAT |
| SIP2pb-2 | CCTATCGGCAAGGGTATGGT |
| SNF4 up-1 | CCATTTGGACTCGCTACCTC |
| SNF4 up-2 | CATTCATTGTTGACCTCCACTAAGTTTGTGAAACAATATCCAGCA |
| SNF4 dn-1 | GGGCAAAGGAATAGAGTAGATGATGACGGTAATGATTTGGAGG |
| SNF4 dn-2 | AAAGATTATGCGTGGAACCC |
| SNF4-N1 | AGCTCGAGACCTTACCGACCTCCACTCT |
| SNF4-N2 | GATCTAGACGAAATGGGCTCACCACAAG |
| SNF4-C1 | AACTCGAGCGCACGTGTTCGAGATAAGT |
| SNF4-C2 | GCTCTAGACCCCATCCAAAAAGAGTAGC |
| SNF4 pb-1 | TGACGGTAATGATTTGGAGG |
| SNF4 pb-2 | CGAAATGGGCTCACCACAAG |
| SAK1 up-1 | TGTTCTTACCCCCAATAAGCG |
| SAK1 up-2 | CATTCATTGTTGACCTCCACTAACTGTTCGGGCAATGTTTTCT |
| SAK1 dn-1 | GGGCAAAGGAATAGAGTAGATGACTCGCCTTATGTATCCCCTG |
| SAK1 dn-2 | ACCCACAAAACGCCTGTTCT |
| SAK1-N1 | GACTCGAGCCAATAAGCGGGTGTGTAAA |
| SAK1-N2 | GCGTCTAGAAATCGAATGAAGACTGCCTTC |
| SAK1-C1 | AAGTCGACACCCCTACCTCTTTCCGCTAC |
| SAK1-C2 | CGTCTAGAACCCATTCCCTGACCAACTTC |
| SAK1 pb-1 | CTCGCCTTATGTATCCCCTG |
| SAK1 pb-2 | AATCGAATGAAGACTGCCTTC |
| TOS3 up-1 | GAACTGTTGTGGAATGGCGG |
| TOS3 up-2 | CATTCATTGTTGACCTCCACTAAGTGGAGAGAGGGGTTTGTGT |
| TOS3 dn-1 | GGGCAAAGGAATAGAGTAGATGATGAGGGTGAGTTGTGTAGCG |
| TOS3 dn-2 | GGCGTGAGGTAGACGTAAGC |
| TOS3-N1 | AACTCGAGATGATGTCGTTGGGCAGTGA |
| TOS3-N2 | CCGTCTAGAGCCTTAAAACTACGAGCCGT |
| TOS3-C1 | ATCTCGAGGGGTAGGGACTTGCCATTCAT |
| TOS3-C2 | GCTCTAGACCCACAAACAAGCCTCTCGC |
| TOS3 pb-1 | TGAGGGTGAGTTGTGTAGCG |
| TOS3 pb-2 | GCCTTAAAACTACGAGCCGT |
| SUR-1 | GTGCCAACGCCACAGTGCC |
| SUR-2 | GTGAGAGCATGCAATTCCCGT |
| TOS3up-2d | TGGGGCACTGTGGCGTTGGCACAGTGGAGAGAGGGGTTTGTGT |
| TOS3dn-1d | CACGGGAATTGCATGCTCTCACATGAGGGTGAGTTGTGTAGCG |
| TOS3-N1d | CGTCTAGAAGGCTGTATCTGACCCGTTTG |
| TOS3-N2d | GACTCGAGACTACGAGCCGTGTTTGTGC |
|  |  |
| **qRT-PCR** | |
| SNF1-QF | TCGGTAATGACGGATCCAAAG |
| SNF1-QR | TCTGTATGGAAAGTCGCATGG |
| SIP2-QF | CTGCTCTTCCTGGTTTCCTC |
| SIP2-QR | GTTCTTGATGCTTGAAGTGGC |
| SNF4-QF | ACCCTCTTCCTACCTTCGTC |
| SNF4-QR | CGTCATAGCTTGTCCTGATCTTC |
| SAK1-QF | GTAGATCGTGACGGTACAACTG |
| SAK1-QR | CGCGGGATAATTTTGATGGC |
| TOS3-QF | CCACGTACCAGAGTCCAATTC |
| TOS3-QR | CTGGTTGACTGTTTGGTGTG |
| PEX1-QF | ATGGTCAAGTGCTCGGAATC |
| PEX1-QR | AGGGATTGGCTTCTGTGATC |
| PEX11-QF | CATCAGACCCTCCAAGAACG |
| PEX11-QR | GATGCTAAAGGTCAAGCCAAAG |
| MFP1-QF | TTCGGTAACTTCGGTCAGTG |
| MFP1-QR | ATCACAGTCTCAGTCATGCG |
| ICL1-QF | TGGAAAGACTGGCGATCAAC |
| ICL1-QR | CGAATGGACGAGGAAGAGTTG |
| β-Tubulin-QF | ACAACTTCGTCTTCGGTCAG |
| β-Tubulin-QR | GTGATCTGGAAACCCTGGAG |
|  | |
| **Yeast two-hybrid** | |
| SNF1-BDF | CATGGAGGCCGAATTCATGGCCCACTGCCAGGACCGAGGC |
| SNF1-BDR | GCAGGTCGACGGATCCCTAGTCAGCATCGGCAAGCTGTAT |
| SIP2-ADF | GGAGGCCAGTGAATTCATGGGCAATCAAACCTCCGCCCAG |
| SIP2-ADR | CGAGCTCGATGGATCCTTAGCCATCTTGACCTGTTGGCTT |
| SIP2-BDF | CATGGAGGCCGAATTCATGGGCAATCAAACCTCCGCCCAG |
| SIP2-BDR | GCAGGTCGACGGATCCTTAGCCATCTTGACCTGTTGGCTT |
| SNF4-ADF | GGAGGCCAGTGAATTCATGGATGATGTGCCTGCAGCTCCG |
| SNF4-ADR | CGAGCTCGATGGATCCTCATTTCCTGCCATCTTTGGAACC |
| SAK1-ADF | GGAGGCCAGTGAATTCATGCGATCCCTTGAACGCTACCGA |
| SAK1-ADR | CGAGCTCGATGGATCCTCAGTTGTGTTGCGAGACTCTTCT |
| TOS3-ADF | GGAGGCCAGTGAATTCATGGAAACGCCTCTCACGCCACCA |
| TOS3-ADR | CGAGCTCGATGGATCCCTACTTAGCGTCCTCTATCGTCAG |
